# Fluorescent Peptides Internalize HeLa Cells and Kill Multidrug-Resistant Clinical Bacterial Isolates

**DOI:** 10.3390/antibiotics14080793

**Published:** 2025-08-04

**Authors:** Daniel Castellar-Almonacid, Kelin Johana Cuero-Amu, Jose David Mendoza-Mendoza, Natalia Ardila-Chantré, Fernando José Chavez-Salazar, Andrea Carolina Barragán-Cárdenas, Jhon Erick Rivera-Monroy, Claudia Parra-Giraldo, Zuly Jenny Rivera-Monroy, Javier García-Castañeda, Ricardo Fierro-Medina

**Affiliations:** 1 Departamento de Farmacia, Facultad de Ciencias, Universidad Nacional de Colombia-Sede Bogotá, Carrera 45 No 26-85, Building 451, Bogotá D.C. 111321, Colombia; dcastellara@unal.edu.co (D.C.-A.); nardilac@unal.edu.co (N.A.-C.); 2 Instituto de Biotecnología, Facultad de Ciencias, Universidad Nacional de Colombia-Sede Bogotá, Carrera 45 No 26-85, Building 451, Bogotá D.C. 111321, Colombia; kcueroa@unal.edu.co (K.J.C.-A.); jomendozam@unal.edu.co (J.D.M.-M.); 3 Departamento de Química, Facultad de Ciencias, Universidad Nacional de Colombia-Sede Bogotá, Carrera 45 No 26-85, Building 451, Bogotá D.C. 111321, Colombia; fchavezs@unal.edu.co (F.J.C.-S.); zjriveram@unal.edu.co (Z.J.R.-M.); rfierrom@unal.edu.co (R.F.-M.); 4 Bacteriología y Laboratorio Clínico, Facultad de Ciencias de la Salud, Universidad Colegio Mayor de Cundinamarca, Bogotá D.C. 110311, Colombia; abarraganc@unal.edu.co; 5 Laboratorio Instrumental de Alta Complejidad, Universidad de La Salle, Carrera 5 No. 59A-44, Bogotá D.C. 110231, Colombia; jhrivera@lasalle.edu.co; 6 Biomedical Sciences Faculty, Universidad Europea, 28670 Madrid, Spain; claudia.parra@universidadeuropea.es

**Keywords:** peptide-based therapy, rhodamine labeling, LfcinB, peptide internalization, cytoplasmic localization, SPPS

## Abstract

Palindromic antimicrobial peptides (PAMs) constitute versatile scaffolds for the design and optimization of anticancer agents with applications in therapy, diagnosis, and/or monitoring. In the present study, fluorolabeled peptides derived from the palindromic sequence RWQWRWQWR containing fluorescent probes, such as 2-Aminobenzoyl, 5(6)-Carboxyfluorescein, and Rhodamine B, were obtained. RP-HPLC analysis revealed that the palindromic peptide conjugated to Rhodamine B (RhB-RWQWRWQWR) exhibited the presence of isomers, likely corresponding to the open-ring and spiro-lactam forms of the fluorescent probe. This equilibrium is dependent on the peptide sequence, as the RP-HPLC analysis of dimeric peptide (RhB-RRWQWR-hF-KKLG)_2_K-Ahx did not reveal the presence of isomers. The antibacterial activity of the fluorescent peptides depends on the probe attached to the sequence and the bacterial strain tested. Notably, some fluorescent peptides showed activity against reference strains as well as sensitive, resistant, and multidrug-resistant clinical isolates of *E. coli*, *S. aureus*, and *E. faecalis*. Fluorolabeled peptides 1-Abz (MIC = 62 µM), RhB-1 (MIC = 62 µM), and Abz-1 (MIC = 31 µM) exhibited significant activity against clinical isolates of *E. coli*, *S. aureus*, and *E. faecalis*, respectively. The RhB-1 (IC_50_ = 61 µM), Abz-1 (IC_50_ = 87 µM), and RhB-2 (IC_50_ = 35 µM) peptides exhibited a rapid, significant, and concentration-dependent cytotoxic effect on HeLa cells, accompanied by morphological changes characteristic of apoptosis. RhB-1 (IC_50_ = 18 µM) peptide also exhibited significant cytotoxic activity against breast cancer cells MCF-7. These conjugates remain valuable for elucidating the possible mechanisms of action of these novel anticancer peptides. Rhodamine-labeled peptides displayed cytotoxicity comparable to that of their unlabeled analogues, suggesting that cellular internalization constitutes a critical early step in their mechanism of action. These findings suggest that cell death induced by both unlabeled and fluorolabeled peptides proceeds predominantly via apoptosis and is likely contingent upon peptide internalization. Functionalization at the N-terminal end of the palindromic sequence can be evaluated to develop systems for transporting non-protein molecules into cancer cells.

## 1. Introduction

Antibiotic resistance is the ability of pathogens to survive exposure to antibiotics to which they were previously sensitive. It arises from the misuse of antibiotics, delayed diagnosis, and the presence of antibiotics in the environment [1]. Antibiotic resistance can be either intrinsic or acquired, involving various genetic mechanisms [1]. Pathogens resistant to one or two classes of antibiotics, as well as those resistant to three or more classes (multiresistant), have been identified [1]. The emergence and spread of drug-resistant pathogens compromise our ability to treat common infections [1]. The clinical development pipeline for new antimicrobials is severely limited. In 2019, there were 32 antibiotics in clinical development with the potential to target pathogens on the WHO priority list, of which only six were classified as innovative [1]. New antibacterials are urgently needed to treat infections caused by Gram-negative bacteria resistant to carbapenem antibiotics included on the WHO list of priority pathogens [1].

On the other hand, cancer comprises a group of pathologies characterized by the uncontrolled proliferation of cells forming tumors in various parts of the body, with the potential to migrate and colonize other tissues (metastasis) [2]. The cell cycle is altered, and cancer cells proliferate uncontrollably due to the inhibition of programmed cell death pathways [3]. Tumor growth demands oxygen and nutrients, inducing angiogenesis and the formation of abnormal blood vessels [4,5]. The altered tumor microenvironment makes it difficult for immune cells to access the tumor, and mechanisms are developed to evade immune surveillance [4,5]. Cancer morbidity and mortality can be reduced through early detection and appropriate treatment [2]. Chemotherapy remains one of the primary treatments for cancer; however, drug resistance often emerges, reducing its efficacy. In vitro studies suggest that multidrug resistance can be transmitted from cancer progenitor cells to their progeny [6].

Cancer patients are at increased risk for infectious diseases compared to healthy individuals. Studies have shown that infections contribute to more than half of cancer mortality and can result in the postponement or suspension of treatment, reducing the likelihood of favorable outcomes [7]. Antimicrobial resistance has been reported more frequently in ambulatory cancer patients than in non-cancer patients, indicating the need for enhanced surveillance, infection prevention, and the development of new drugs with both antimicrobial and anticancer activity [8]. Bacteria and cancer cells utilize mechanisms to evade cell death. Bacteria migrate away from the cytotoxic microenvironment, resembling tumor metastasis [4,5,6]. Biofilm formation creates a protective barrier, while the altered tumor microenvironment supports tumor progression [4,5,6]. Studies of residual cancer cells and bacterial persisters reveal striking behavioral parallels, underscoring the urgent need to refine therapeutic strategies targeting these drug-tolerant cells (DTCs) in both oncology and bacteriology [9].

Studies have shown that bacteria can be internalized by cancer cells, although it remains unclear whether tumor-associated bacteria influence tumorigenesis, cancer progression, or metastasis [10,11]. In a murine model of breast cancer, removal of intracellular bacteria resulted in reduced lung metastasis, suggesting that bacteria may influence the metastatic process rather than primary tumor growth [10,11]. *E. coli* strains isolated from breast cancer patients induce DNA double-strand breaks in cancer cells, promoting carcinogenesis and increasing proliferation and invasiveness. Chronic pulmonary infections with *P. aeruginosa* and *S. aureus* have been associated with enhanced lung metastasis of breast cancer in mice [12].

Methods for cancer diagnosis have been developed, including fluorescent cell imaging, biomarker identification via LC-MS, and automated DNA sequencing [13,14]. Fluorescent antimicrobial peptides (FAMPs) are powerful tools for studying the mode of action of these molecules and their peptide–bacteria interactions [15]. Diagnosis, treatment monitoring, and image-guided surgery using fluorescent probes are widely used due to their high sensitivity, real-time analysis, and relative simplicity [16,17]. Unlike normal cells, bacterial and cancer cell membranes possess a net negative charge due to the presence of glycan groups. Therefore, cationic peptides with antimicrobial (AMPs) and anticancer (APCs) activity are of interest due to their selectivity for bacterial and cancer cells, without affecting normal cells [18,19,20]. Peptides emerge as a valuable alternative for the identification and development of new drugs for the treatment and diagnosis of diseases [18,19,20,21]. AMPs and ACPs are considered a broad and diverse source for the design, development, and optimization of promising candidates for preclinical and clinical studies [21].

Fluorolabeled peptides represent an attractive alternative for the development of fluorescent probes for diagnostics, therapy, and research [22,23]. Synthetic peptides offer multiple advantages, including ease of design, synthesis, and optimization, enabling the generation of probes with specificity for cellular targets, improved ADME properties, biocompatibility, self-assembly capacity, and cost, among others [17,22]. Fluorolabeled probes can be designed using AMPs and ACPs that interact specifically with membrane, cytoplasmic, and nuclear molecules, enabling their application in diagnosis, treatment, and mechanism studies [23,24,25]. The fluorescent labeling of peptides and proteins is a critical step, requiring careful consideration of the probe type, peptide sequence, probe-binding site, chemical bond type, yield, and purity [15]. Importantly, the incorporation of the fluorophore must not compromise the activity, selectivity, or toxicity of the molecule [26]. Most fluorophores contain aromatic rings, making them bulky, rigid, and hydrophobic, potentially altering the peptides’ anticancer activity, selectivity, toxicity, and mechanism of action [26].

Short peptides derived from bovine lactoferricin (LfcinB) have shown antimicrobial activity against both Gram-positive and Gram-negative bacteria and selective, rapid, and sustained anticancer activity in vitro against various cancer cell lines (MCF-7, MDA-MB-231, MDA-MB-431, HeLa, Ca Ski, Caco 2, HT29, etc.) [27,28,29,30]. The palindromic peptide RWQWRWQWR (referred to here as peptide 1) has shown antimicrobial activity against reference and clinical strains (sensitive, resistant, and multiresistant) of bacteria (*E. coli*, *S. aureus*, etc.) and fungi (*C. albicans* and *C. auris*) [31]. This peptide is considered a broad-spectrum anticancer peptide due to its selective cytotoxicity against breast, colon, and cervical cancer cell lines [27,28,29]. It induces morphological changes such as cell rounding, shrinkage, and activation of caspases. This peptide induced mitochondrial membrane depolarization, caspases activation, and cell population in early and late apoptosis [27,28,29]. Similarly, the dimeric peptide (RRWQWR-hF-KKLG)_2_K-Ahx (referred to as peptide 2) exhibited a significant cytotoxic effect against MCF-7 and HeLa cell lines [29]. Both peptides displayed cancer cell selectivity, and their cytotoxicity appears to be mediated through apoptotic pathways [27,28,29]. However, it remains unclear whether peptides 1 and 2 interact with specific membrane targets to induce cell death or are internalized to act on cytoplasmic or nuclear components.

In this study, peptides 1 and 2 were labeled with various fluorescent molecules to assess whether incorporation of the fluorescent motif in the sequence affects anticancer and antibacterial activity. Our findings indicated that the antibacterial and cytotoxic activity of fluorolabeled peptides is influenced by the primary structure of the peptide, the position of the probe attachment, and the chemical nature of the fluorophore. Cells treated with RhB-labeled peptides exhibited fluorescence localized in both the cytoplasm and nucleus of HeLa cells, suggesting peptide internalization and potential interactions with intracellular targets. These results underscore the utility of SPPS-Fmoc/*t*Bu for synthesizing fluorolabeled peptides. Depending on the probe and peptide sequence, it is possible to obtain conjugates with equal or enhanced anticancer and antibacterial activity, which can be used to determine cellular localization and the mechanism of action.

## 2. Results

### 2.1. Synthesis of Peptides

Three fluorescent probes, 2-aminobenzoyl (Abz), 5(6)-carboxyfluorescein (5(6)-FAM), and Rhodamine B (RhB), were incorporated into the palindromic peptide sequence to assess their effects on synthetic yield, the LC–MS profile, and both antibacterial and anticancer activities. Using Fmoc/*t*Bu solid-phase peptide synthesis (SPPS), all fluorolabeled analogues were obtained in a high yield regardless of probe size, shape, structure, or attachment site within the peptide chain (Table 1). LC–MS analysis confirmed complete coupling of each probe, indicating that TBTU/DIPEA-mediated amide bond formation proceeded with high efficiency (Table 1).

All peptides were obtained by SPPS-Fmoc/*t*Bu and purified by RP-SPE chromatography, and the chromatographic profiles of pure products showed a single major peak with purities greater than 88% and molecular weights corresponding to the expected values (Figures S1–S11, Supplementary Materials; Table 1). The chromatographic profile of the crude peptide Abz-1 showed a single peak, which corresponded with the expected product, allowing isolation of the peptide with high purity (89.7%). In contrast, the crude peptide 1-Abz displayed three main species with retention times (t_R_) of 7.3 min, 7.8 min, and 8.7 min. The species at t_R_ = 7.3 min corresponded to the expected molecule (peptide 1-Abz), while the other species were identified as undesired products (Table 1 and Figures S1 and S2, Supplementary Materials).

The crude product of the FAM-1 peptide exhibited multiple peaks, with two major species at t_R_ = 7.8 and 8.2 min, with the latter corresponding to the expected product. The conjugation of 5(6)-carboxyfluorescein to the palindromic sequence 1 resulted in a product (FAM-1) composed of a mixture of several species. The chromatographic profile revealed a species with t_R_ = 8.2 min, corresponding to the expected peptide (Table 1 and Figure S3, Supplementary Materials). The species at t_R_ = 7.0 min may be a degradation product of the fluorescent core generated during synthesis, as its mass did not suggest amino acid deletions, indicating the peptide sequence remained intact. However, MS data did not allow for the proposal of a plausible structure for this product. The FAM-1 peptide was obtained with a purity greater than 94%, and it was established that the pure product consists of a mixture of the 5- and 6-carboxyfluorescein positional isomers (Table 1 and Figure S3, Supplementary Materials).

**Figure 1:**
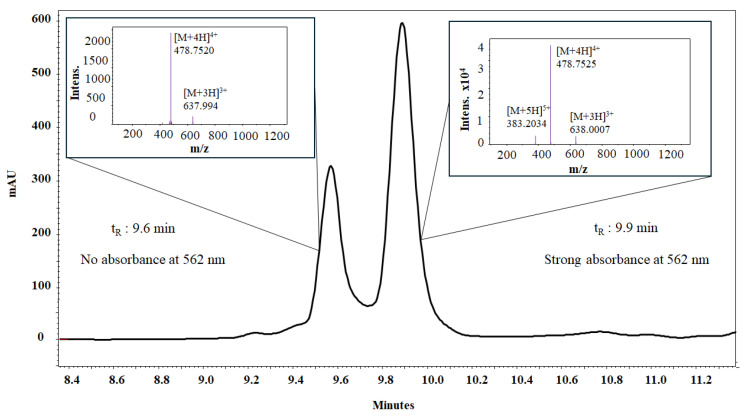
RP-HPLC and ESI-QTOF analysis of peptide RhB-1. An equilibrium between isomeric forms of Rhodamine-labeled peptide RhB-1 can be followed by liquid chromatography. The RP-HPLC chromatogram shows two resolved species with identical monoisotopic mass, as confirmed by ESI-QTOF mass spectra for both peaks.

The conjugation of Rhodamine to the palindromic sequence yielded a product that showed two peaks in the RP-HPLC analysis, with retention times of t_R_ = 9.6 min and t_R_ = 9.9 min. The masses of both species, calculated from the [M+4H]^4+^ ion and the isotopic distribution of the base peak, coincided, suggesting the presence of a mixture of two isomers. The species observed in the chromatogram correspond to the spiro-lactam (t_R_ = 9.6 min) and open-ring forms (t_R_ = 9.9 min) (Figure 1 and Figures S5–S8, Supplementary Materials). The open-ring isomer adsorbs electromagnetic radiation at 562 nm, whereas the spiro-lactam form does not absorb in the visible region (Figure S6, Supplementary Materials).

The incorporation of the fluorescent probes into the palindromic sequence increased the retention times, indicating an increase in hydrophobicity in the following order: 1 < Abz-1 ≈ 1-Abz < FAM-1 < RhB-1 (Table 1). All fluorolabeled peptides were water soluble, enabling their characterization, purification, and subsequent evaluation of biological activity.

### 2.2. Antibacterial Activity of the Conjugates in Reference Strains

The antibacterial activity of peptides was evaluated against reference strains of Gram-positive and Gram-negative bacteria. Peptide 1 showed activity against *E. coli* ATCC 25922, *K. pneumoniae* ATCC 700603, and *S. aureus* ATCC 25923 strains. Peptides 1, 1-Abz, and Abz-1 exhibited antibacterial activity against *E. coli* ATCC 25922, while peptides RhB-1 and FAM-1 demonstrated lower antibacterial activity in this strain.

Peptide Abz-1 exhibited antibacterial activity in *E. coli* ATCC 25922, *S. aureus* ATCC 25923, and *E. faecalis* ATCC 29212, while peptide 1-Abz was active against *E. coli* ATCC 25922 and *S. aureus* ATCC 25923. The peptides RhB-1, 1-Abz, Abz-1, and FAM-1 did not exhibit antibacterial activity against *P. aeruginosa* and *S. aureus* reference strains. Peptide FAM-1 did not show antibacterial activity against any of the tested strains. Peptide RhB-1 showed activity against the Gram-positive strains *S. aureus* ATCC 25923 and *E. faecalis* ATCC 29212 (Table 2).

### 2.3. Characterization and Susceptibility Profile of Clinical Isolates

Based on the results of fluoresced peptides in the reference strains, *E. coli*, *S. aureus*, and *E. faecalis* bacteria were selected for evaluation in susceptible, resistant, and multidrug-resistant (MDR) clinical isolates. The isolates were obtained from the National Cancer Institute collection and were initially identified and antibiogrammed using the VITEK system to determine their antibacterial susceptibility profiles (Table S1, Supplementary Materials).

*E. coli* isolates 1004 (susceptible), 129797 (resistant), and 301755 (MDR) were selected for evaluation. Isolate 1004 was sensitive to all antibiotics tested. Clinical isolate 129797 exhibited resistance to ampicillin, cephalothin, and the ampicillin/sulbactam combination, indicating it is a cephalosporinase-producing strain. This type of beta-lactamase enables hydrolysis of both penicillins and first-generation cephalosporins and shows that sulbactam is ineffective as an inhibitor. In contrast, clinical isolate 301755 demonstrated resistance to penicillins (ampicillin and ampicillin/sulbactam), third- and fourth-generation cephalosporins (ceftriaxone, ceftazidime, cefepime), aminoglycosides (gentamicin), fluoroquinolones (norfloxacin and ciprofloxacin), nitrofurans (nitrofurantoin), and diaminopyrimidines (trimethoprim/sulfamethoxazole) (Table S1). These findings identify it as a multidrug-resistant (MDR) strain and an extended-spectrum beta-lactamase (ESBL)-producing bacterium. For *S. aureus*, isolates 109095, 117719, and 124653 were evaluated. All three were resistant to penicillin, indicating beta-lactamase production mediated by the *blaZ* gene. Studies have shown that up to 90% of *S. aureus* isolates worldwide are penicillin-resistant [32], which implies that this antibiotic is ineffective in infections caused by this pathogen. Isolate 117719 also exhibited resistance to tetracycline, which inhibits bacterial protein synthesis via the 30S ribosomal subunit. Isolate 124653 was resistant to penicillin, tetracycline, and erythromycin, with the latter belonging to the group of macrolides, antibiotics that inhibit protein synthesis through interaction with the 50S rRNA subunit. For *E. faecalis* three isolates were selected: strains 213 (sensitive to all the antibiotics tested), strain 82 (resistant to streptomycin), and strain 179 (resistant to gentamicin). These clinical isolates were selected to assess the antimicrobial activity of the peptide RWQWRWQWR and its analogues conjugated to fluorescent molecules in strains against strains with susceptibility profiles.

### 2.4. Antibacterial Activity Against Isolates of E. coli, S. aureus, and E. faecalis

To determine whether the peptides possess antibacterial activity against sensitive, resistant, or multidrug-resistant clinical isolates of *E. coli*, several conjugates were tested. Peptide 1 exhibited significant antibacterial activity against all three *E. coli* clinical isolates evaluated. Peptide 1-Abz also showed antibacterial activity against these isolates, although to a lesser extent than peptide 1 (Table 3).

Antibacterial assays against *S. aureus* clinical isolates showed that only peptide RhB-1 exhibited significant antibacterial activity against all three clinical isolates evaluated. Peptides 1, 1-Abz, Abz-1, and FAM-1 did not show antibacterial activity against the *S. aureus* clinical isolates (Table 3).

On the other hand, peptides 1 and Abz-1 showed activity against all three *E. faecalis* clinical isolates, with Abz-1 showing the highest antibacterial activity among them. Peptides RhB-1 and FAM-1 also showed activity against the resistant clinical isolate 82 (Table 3).

### 2.5. Rhodamine B Conjugation Increases Cytotoxic Activity of Palindromic and Dimeric Peptides

MTT assays were conducted in HeLa cells to evaluate the effect of fluorescent probe incorporation on the in vitro cytotoxic activity of the parent non-conjugated peptides. The cytotoxic effect of peptides 1, Abz-1 RhB-1, 2, and RhB-2 in HeLa cells was peptide concentration-dependent, with the highest anticancer activity observed at 200 µg/mL. Incorporation of 2-Abz and RhB at the N-terminal end reduced cytotoxic activity, while the binding of 2-Abz at the C-terminal end or FAM at the N-terminal end drastically diminished anticancer activity (Table 4).

**Figure 2:**
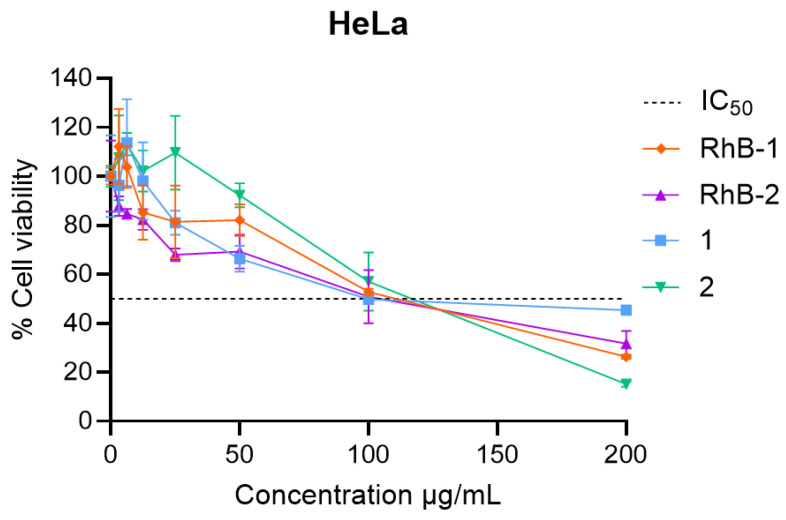
Cell viability plots in HeLa cells of the RhB-conjugated peptides compared to their non-conjugated parent peptides. Experiments were conducted in triplicate (*n* = 3). Data are shown as mean ± S.D. Two-way ANOVA and Sidak’s multiple comparison test were performed (*p* < 0.05). No statistically significant differences were observed between RhB-1 and peptide 1 across the tested concentration range. RhB-2 showed statistically significant differences compared to peptide 2 at 100 and 200 µg/mL.

For the palindromic peptide, the inclusion of RhB decreased the IC_50_ value, although no significant statistical differences in activity were observed in HeLa cells within the evaluated concentration range for both peptides. Therefore, incorporation of the fluorescent probe does not significantly affect the in vitro anticancer activity of peptide 1 (Figure 2). In contrast, for the dimeric peptide 2, the inclusion of Rhodamine B did not alter the IC_50_ value, indicating similar cytotoxic activity (Table 4). However, RhB-2 exhibited a higher cytotoxic effect than peptide 2 at concentrations between 0 and 100 µg/mL, while at 200 μg/mL, cell viability was approximately 40% for RhB-2, compared to about 20% for the palindromic peptide (Figure 2).

**Figure 3:**
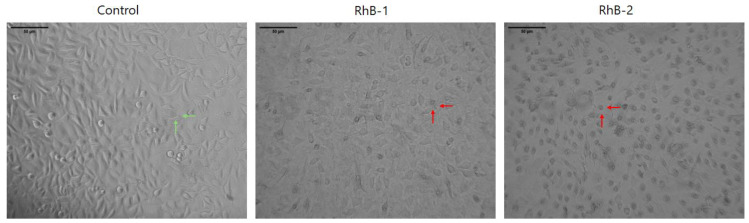
Micrographs of HeLa cells after a 2 h treatment with RhB-1 or RhB-2 (100 µg/mL) at 37 °C. Green arrows indicate normal morphologies, such as cytoplasmic elongations and polygonal shapes. Red arrows indicate morphological changes induced by the peptides, including cell rounding, shrinkage, and loss of cytoplasmic projections. Scale bar: µm. The control corresponds to untreated cells.

Cells treated with peptides RhB-1 and RhB-2 at 100 µg/mL for 2 h exhibited significant morphological changes compared to untreated cells. Microphotographs revealed that cells exposed to RhB-1 and RhB-2 appeared smaller, rounded, and contained vacuoles. Despite these pronounced morphological alterations, there was no evidence of cell rupture, membrane disintegration, or the presence of cell fragments, indicating the absence of necrotic processes. In contrast, untreated control cells displayed normal morphology, including cytoplasmic extensions, an elongated shape, and no observable vacuoles (Figure 3).

### 2.6. Rhb-1 and RhB-2 Peptides Are Localized in the Nucleus and Cytoplasm of HeLa Cells

**Figure 4:**
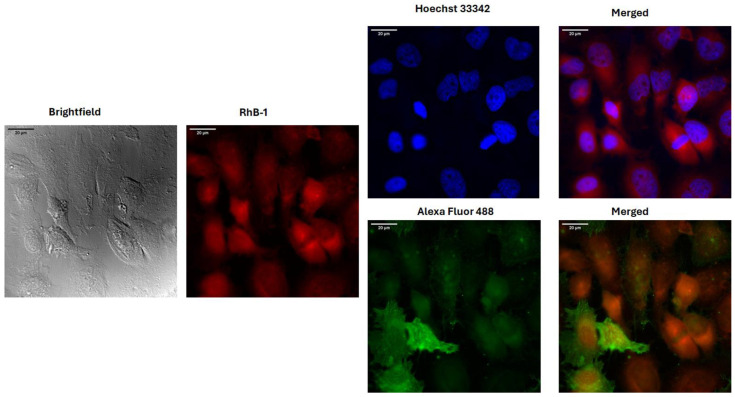
Fluorescence confocal microscopy images of HeLa cells treated for two hours with RhB-1 at its respective IC_50_. Stains for the nucleus (Hoechst 3322) and cytoplasmic actin (Alexa Fluor 488 alpha SMA) were employed for determining colocalization. Red: RhB-1 fluorescence; Blue: Hoechst 33242 nuclear stain; Green: Alexa Fluor 488 alpha SMA actin stain.

Fluorescence confocal microscopy imaging of HeLa cells treated for 2 h with the IC_50_ concentration of labeled peptides revealed colocalization with the cells, confirming physical interaction. The SMA antibody labeled with Alexa Fluor 488 was used to stain proteins such as cytoplasmic actin, while Hoechst 33342 was employed to visualize the cell nucleus through its interaction with DNA. In the microphotographs, colocalization of the Rhodamine B-labeled peptides (red) with the HeLa cells is observed, and the cell nucleus is clearly distinguishable. Cells treated with RhB-1 and RhB-2 peptides remained intact, exhibiting morphological changes similar to those previously observed, including cell rounding and shrinkage (Figure 4).

### 2.7. Cytotoxic Effect of RhB-1 Peptide in MCF-7 Cells

**Figure 5:**
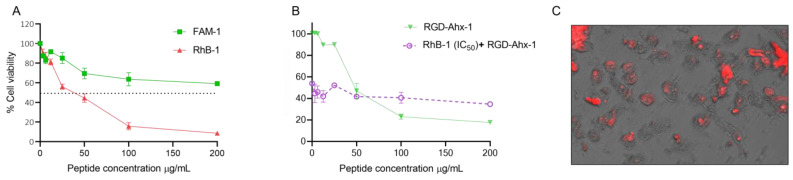
(**A**) Cell viability curves of MCF-7 cells treated with FAM-1 and RhB-1 peptides for 2 h at 37 °C. (**B**) Competition assay in MCF-7 cells stimulated for 2 h with the IC_50_ (34 μg/mL) of the RhB-1 peptide, followed by treatment with the RGD-Ahx-1 peptide at concentrations from 0 to 200 μg/mL (red). (**C**) Fluorescence microphotographs of cells after treatment with the RhB-1 peptide. Dashed line indicates the IC_50_ (50% cell viability). Experiments were conducted in triplicate (*n* = 3). Data is shown as mean ± S.D. Two-way ANOVA and Sidak’s multiple comparisons tests were performed (*p* < 0.05).

An MTT assay was performed in MCF-7 cells with FAM-1 (FAM-RWQWRWQWR) and RhB-1 (RhB-RWQWRWQWR) peptides following a 2 h treatment. The results demonstrated that the addition of fluorochromes to the palindromic sequence preserved its concentration-dependent cytotoxic activity, although this activity was significantly reduced when the palindromic sequence was conjugated with fluorescein (Figure 5A). The cytotoxic effect of the FAM-1 peptide was limited, affecting only 41% of the cell population at the maximum concentration (200 µg/mL), and thus, the IC_50_ value could not be determined. In contrast, the RhB-1 peptide showed significant cytotoxic activity in MCF-7 cells (IC_50_ = 34 µg/mL; 18 µM), rapidly and dramatically reducing the cell population to 8% at 200 µg/mL (Figure 5A).

The RGD-Ahx-1 peptide (RGD-Ahx-RWQWRWQWR) showed a significant and selective cytotoxic effect in MCF-7 cells (IC_50_ = 27 μg/mL; 14 µM) [33]. Both RGD-AHx-1 and RhB-1 peptides exhibited greater cytotoxic effects than the unfunctionalized palindromic peptide (RWQWRWQWR). Previous studies also established that RGD-Ahx-1 induces apoptosis-mediated cell death in MCF-7 cells. A preliminary competition assay was performed between RhB-1 and RGD-Ahx-1 peptides: cells were treated with RhB-1 (34 μg/mL) for 2 h at 37 °C, followed by the addition of RGD-Ahx-1 at final concentrations of 0–200 μg/mL (Figure 5B, red). As a control, cells were treated with RGD-Ahx-1 alone at the same concentrations for 4 h at 37 °C (Figure 5B, green). The cytotoxic activity was reduced in cells pretreated with RhB-1 compared to those treated only with RGD-Ahx-1. Fluorescence microscopy images confirmed that MCF-7 cells were stained following RhB-1 peptide treatment (Figure 5C).

### 2.8. Hemolytic Activity of Fluorolabeled Peptides

Peptide 1 (RWQWRWQWR) showed 4.5% hemolysis at a concentration of 200 µg/mL, classifying it as non-hemolytic (<10% hemolysis) with a therapeutic index (TI) greater than eight. In contrast, the fluorolabeled analogues FAM-1 and RhB-1 induced 20.9% and 42.4% hemolysis, respectively, at their highest tested concentrations, corresponding to low selectivity (TI of 0.25 and 0.5, respectively). The N-terminally labeled Abz-1 (2-aminobenzoic acid) caused 13.4% hemolysis at the highest concentration evaluated; however, its therapeutic index was four, indicating that the peptide retains selectivity, since the therapeutic index is greater than one. Interestingly, C-terminal attachment of 2-aminobenzoic acid in 1-Abz reduced hemolysis to 1.5% at 200 µg/mL while preserving a TI of four, demonstrating that C-terminal fluorolabeling enhances hemocompatibility without compromising peptide selectivity.

## 3. Discussion

In this study, the palindromic sequence RWQWRWRWQWR was fluorolabeled with 2-Abz, FAM, and RhB fluorescent probes to assess synthetic viability and evaluate the effect of fluorophore incorporation on antibacterial and anticancer activity.

### 3.1. Fluorolabeled Peptides Synthesis and Characterization

Initially, fluorolabeled peptides incorporating the 2-ABz probe at the N- and C-terminal were synthetized, as 2-Abz is the smallest of the three probes. Peptides (2-Abz)-1 and 1-(2-Abz) were successfully synthesized with high purity, and their experimental masses matched expected values. Incorporation at the N-terminus proved more synthetically efficient, requiring only one coupling reaction (Figures S1 and S2, Supplementary Materials) and yielding a product with a single dominant chromatographic species. In contrast, the C-terminal conjugate 1-Abz crude product yielded multiple peaks, with the desired product at 7.3 min (MW = 1604.7980) and two major by-products at 7.8 min (MW = 1117.2537) and 8.7 min (MW = 1448.6970), corresponding to peptides with deletions, with the 8.7 min species being the most abundant (Figures S1 and S2, Supplementary Materials). This indicates that the position of fluorophore attachment influences synthesis efficiency, with N-terminal incorporation being more favorable.

The FAM-1 peptide was obtained with chromatographic purity greater than 94% and the molecular mass (1843.8112 u) corresponds with that expected, and it was established that the pure product consists of a mixture of the 5- and 6-carboxyfluorescein positional isomers (Table 1 and Figure S3, Supplementary Materials). To establish if the peptide FAM-1 coeluted with impurities, the peak purity was determined using the DAD detector; the results showed that the pure product showed a peak purity of 98.9% (Figure S4, Supplementary Materials).

The chromatographic profile of the crude RhB-1 peptide showed two main peaks (t_R_ = 9.6 min and 9.9 min), and the peptide was purified by RP-SPE chromatography (Figure S12A), corresponding to the spiro-lactam and open-ring isomers, respectively (Figure S12A). A chromatographic method was developed to resolve the two peaks, and aliquots of each were collected from the HPLC. The fraction corresponding to the spiro-lactam form (t_R_ = 8.4 min) was initially colorless, whereas the fraction corresponding to the open-ring form (t_R_ = 8.7 min) was pink. Interestingly, the colorless fraction gradually turned pink over time. Both fractions were individually analyzed by HPLC at 15 min and again 12 h post-collection (Figure S12A). The chromatograms of both samples displayed the two peaks, and the 15 min analyses exhibited different peak ratios compared to the 12 h analyses. Furthermore, the peptide was dissolved in aqueous buffers at pH 2.0, 6.0, and 9.0, and its absorbance spectrum was recorded from 200 to 700 nm for each condition. At pH 2.0, the pink open-ring form predominated, but its relative abundance decreased as the pH increased; by pH 9.0 the solution was colorless, indicating predominance of the spiro-lactam form (Figure S12B). These observations are consistent with previous reports demonstrating that the spiro-lactam and open-ring forms exist in a pH-dependent equilibrium, indicating that purified samples invariably comprise a mixture of both isomeric species [34].

**Figure 6:**
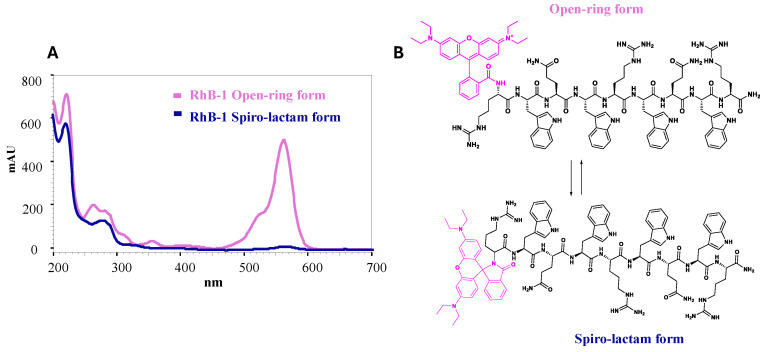
Isomerism of RhB-1 peptide. (**A**) UV–VIS spectra of the spiro-lactam (t_R_ = 9.6 min, blue) and open-ring (t_R_ = 9.9 min, pink) forms of the RhB-1 peptide. (**B**) Schematic of the equilibrium of the proposed equilibrium between the isomeric forms of RhB-1. Magenta: Rhodamine B (RhB) moiety.

In contrast, RhB-1 peptide, after purification by RP-SPE, displayed two closely eluting peaks at t_R_ = 9.6 min and 9.9 min. LC-MS analysis confirmed identical mass spectra and isotopic distributions (Figure 1 and Figures S4–S8, Supplementary Materials), suggesting these peaks correspond to structural isomers. Despite multiple purification attempts, the isomers could not be separated; re-injection of individually collected peaks showed re-equilibration to the same chromatographic profile and molecular weight calculated from mass spectra (Figures S7 and S8, Supplementary Materials). To further explore the possible nature of this isomeric pair, DAD analysis was performed in the UV–VIS wavelength range (200 to 700 nm) revealed that only the later-eluting isomer (t_R_ = 9.9 min) absorbed at 562 nm, the characteristic absorption band of Rhodamine B, whereas the earlier peak (t_R_ = 9.6 min) lacked this feature (Table S2 and Figures S5 and S6, Supplementary Materials). These results suggest that the isomers correspond to the non-fluorescent spiro-lactam of the Rhodamine B moiety, which could form a heterocyclic structure with the N-terminal nitrogen of the Arginine residue in peptide 1 (Figure 6). In that regard, the second isomer (t_R_ = 9.9 min) corresponds to the open-ring form, which is fluorescent (Figure S8, Supplementary Materials). A similar equilibrium has been previously observed in peptide–Rhodamine B conjugates, where the presence of an isomer that does not absorb in the visible, but has the same mass as the analogue that does absorb, was evidenced [35].

The presence of a primary amine in the Arginine residue could explain the cyclization of the open-ring form, a process that is pH-dependent and more favorable under basic conditions [34]. The mobile phase employed for chromatographic separation had a pH of 2.0; thus, although acidic conditions favor the open-ring form, a significant proportion of the spiro-lactam isomer was still observed. Given that cell imaging is performed in media at neutral or slightly acidic pH (6.8–7.0), the presence of the non-fluorescent spiro-lactam form of Rhodamine B could potentially compromise fluorescence sensitivity. Nevertheless, the RhB-1 peptide was subsequently tested in in vitro cytotoxic assays and fluorescent microscopy to evaluate its potential as a bioanalytical tool and to have a first approach for the localization in HeLa cells.

Interestingly, RP-HPLC analysis of the dimeric RhB-2 peptide did not show the presence of these isomers (Figures S9–S11, Supplementary Materials). Rhodamine B is conjugated in the dimeric peptide via the same Arginine residues; the spiro-lactam form is not evident in the chromatogram, suggesting that the peptide sequence and molecular structure also influenced this equilibrium between open-ring and spiro-lactam forms. A possible explanation lies in the more complex structure of the RhB-2 peptide, which may be more sterically constrained and could prevent the formation of the spiro-lactam form, at least under acidic pH conditions. Thus, the chromatogram of the dimeric peptide exhibits a major species with a characteristic absorbance at 562 nm (Figures S11 and S13, Supplementary Materials). DAD analysis showed consistent absorption at 562 nm for RhB-2, with peak purity analysis indicating that the major species corresponds predominantly to the fluorescent form (95% purity) (Table S2, Figure S13, Supplementary Materials). These findings represent one of the few cases where equilibrium between Rhodamine B isomers is traceable at low pH using HPLC [34].

### 3.2. Characterization and Susceptibility Profiles of Clinical Isolates

The efficiency of fluorolabeled peptide synthesis by Fmoc/*t*Bu SPPS depends on the chemical structure of the fluorescent probe and generally proceeds smoothly. Isomer formation reflects unavoidable side reactions driven by probe-specific conformational equilibria. Consequently, comprehensive physicochemical characterization of each fluorolabeled peptide is essential to establish its composition, purity, and suitability for downstream applications.

Antibacterial activity of the fluorolabeled peptides was evaluated against clinical isolates *E. coli*, *S. aureus*, and *E. faecalis*, chosen to represent diverse antibiotic-susceptibility phenotypes. Although E. coli is inherently susceptible to most antibiotics, it readily acquires resistance, particularly via horizontal gene transfer, rendering extended-spectrum β-lactamase (ESBL)-producing strains a major public health concern. These ESBL-producers exhibit broad resistance, correlate with poor clinical outcomes, and occur in up to 42.4% of human and 63.2% of veterinary isolates [36,37], underscoring the imperative for novel therapeutics.

*S. aureus* is the most prevalent Gram-positive microorganism in healthcare-associated infections globally (12%) and is classified among the ESKAPE pathogens due to its high resistance potential [32]. Studies have shown that up to 90% of *S. aureus* isolates worldwide are penicillin-resistant [32], which implies that this antibiotic is ineffective in infections caused by this pathogen. Tetracycline resistance in *S. aureus* is primarily mediated by active antibiotic efflux via MFS class proton pumps and ribosome protection via antibiotic cleavage mediated by proteins such as Tet(M) and Tet(S). The main mechanism of *S. aureus* resistance reported for macrolides is the modification of the site of action by the adenylyl-N-methyltransferase Erm enzyme [38]. Finally, *E. faecalis* is an opportunistic pathogen associated with healthcare-related infections and poses a high-risk threat due to increasing antimicrobial resistance. Resistance to aminoglycosides such as gentamicin and streptomycin in *Enterococcus* spp. may be intrinsic or acquired through the transfer of resistance genes. Mechanisms include decreased cell wall permeability, ribosomal mutations, and the production of aminoglycoside-modifying enzymes [39].

### 3.3. Antibacterial Activity

The antibacterial activity of peptides conjugated with fluorescent molecules was evaluated to determine their antibacterial potential, possible utility as tools for studying mechanisms of action and localization of the peptide in the cell, and their potential application in the in vivo monitoring and diagnosis of infectious diseases [25]. For this purpose, bacteria *E. coli* ATCC 25922, *K. pneumoniae* ATCC 700603, *P. aeruginosa* ATCC 27853, *S. aureus* ATCC 25923, and *E. faecalis* ATCC 29212 were selected, several of which belong to the ESKAPE group of pathogens (*E. faecium*, *S. aureus*, *K. pneumoniae*, *A. baumannii*, *P. aeruginosa*, *Enterobacter* spp.), microorganisms of major public health concern due to their high levels of multidrug resistance and significant contribution to global morbidity and mortality [40].

The base peptide RWQWRWQWR, used for fluorescent labeling, showed antibacterial activity against all tested strains, with MICs between 17 and 135 µM (Table 2), indicating that it is a broad-spectrum activity against both Gram-positive and Gram-negative bacteria. These results correlate with previous reports demonstrating the antibacterial activity of this peptide against ATCC strains of *E. coli*, *E. faecalis*, *P. aeruginosa*, and *S. aureus* [41]. The amphipathic structure of the RWQWRWQWR facilitates electrostatic interaction between positively charged residues, such as Arg, with anionic bacterial membrane components, such as lipopolysaccharides in Gram-negative bacteria and teichoic and lipoteichoic acids in Gram-positive bacteria, as well as the hydrophobic interactions between Trp residues with negatively charged phospholipids of the bacterial membrane [42].

The peptide exhibited the highest activity against *E. coli* ATCC 25922 and *S. aureus* ATCC 25923 (MIC = 17µM), suggesting that structural differences in bacterial cell walls between Gram-positive and Gram-negative strains do not impact activity. In *K. pneumoniae* ATCC 700603 and *P. aeruginosa* ATCC 27853 strains, MICs of 67 µM were observed, indicating good antibacterial activity. The difference in antibacterial activity between *E. coli* ATCC 25922 and *K. pneumoniae* ATCC 700603 (4-fold lower activity) may be due to the presence of an extracellular polysaccharide capsule in *K. pneumoniae* strains, a virulence factor that acts as a physical barrier preventing drug interaction with the bacterial cell and allowing evasion of the host’s immune system [43]. Considering that the main mechanism of action proposed for AMPs is the disruption of the cell membrane, the presence of a capsule may prevent interaction with this target, which makes very few peptides active against *K. pneumoniae* strains; however, as observed here with the peptide RWQWRWQWR, some peptides can interact with the capsule, generating its aggregation and disruption, thus exposing the external membrane of the bacteria and ultimately generating cell disruption [44].

In *P. aeruginosa* ATCC 27853, the difference in the antibacterial activity of the RWQWRWQWR peptide with respect to *E. coli* ATCC 25922 may be due to virulence factors, such as the formation of biofilms, where the secretion of extracellular polysaccharides generates a barrier that prevents the interaction of drugs with the bacterial cell [45]. Trp-containing peptides have been known to inhibit virulence factors, such as elastases, proteases, and pyoverdine, as well as suppress gene expression of systems related to bacterial communication that allow the formation of biofilms [46]. These non-bactericidal mechanisms correlate with those observed with the peptide RWQWRWQWR, where an MIC of 67 µM was observed and an MBC > 135 µM, indicating that the peptide has a bacteriostatic effect (Table 2).

The lowest activity was observed in *E. faecalis* ATCC 29212 (MIC = 135 µM; MBC > 135 µM), contrasting with *S. aureus* ATCC 25923 (MIC = 17 µM; MBC = 34 µM). This may be due to changes in the composition of phospholipids in the membranes of these two bacteria; the membrane of Enterococcus spp. is composed of 34% phosphatidylglycerol (PG), 39% cardiolipin (CL), and 27% other phospholipids, while the membrane of *S. aureus* is composed of 50–65% PG, 22% cardiolipin, and 30% lysyl phosphatidylglycerol; these differences have been shown to be key to the effect of peptides such as daptomycin, since the interaction with the negatively charged PG is key to the antibacterial action of AMPs [47,48].

Conjugation with fluorescent molecules diminished activity in *K. pneumoniae* ATCC 700603 and *P. aeruginosa* ATCC 27853. This could be because the conjugated molecules represent a bulky hydrophobic motif that could hinder the interaction with the bacterial cell and, as mentioned above, the presence of virulence factors, such as the capsule in *K. pneumoniae* and the formation of biofilms in *P. aeruginosa*, can prevent the interaction with the cell membrane and generate the loss of the activity of these conjugated peptides [43,45]. Interestingly, RhB-RWQWRWQWR lost activity against Gram-negative strains, while it retained or improved activity against Gram-positive strains. Antibacterial action against Gram-negative bacteria typically requires a peptide net charge of +5 to +9 to disrupt the LPS-rich outer membrane. Gram-positive activity, on the other hand, favors lower net charges and high hydrophobicity (>40%). RhB-RWQWRWQWR, with its increased hydrophobicity (t_R_ = 9.9 min), likely favors interaction with Gram-positive membranes but not with Gram-negatives [48].

Fluorescent labeling with FAM or 2-Abz at the N- and C-terminus preserved activity against *E. coli* ATCC 25922, *S. aureus* ATCC 25923, and *E. faecalis* ATCC 29212, suggesting that these modifications do not hinder interaction with either Gram-positive or Gram-negative membranes. Notably, all labeled peptides showed enhanced activity against *E. faecalis* ATCC 29212, indicating that increased peptide hydrophobicity is crucial for biological activity in this bacterium (Table 2).

These findings suggest that fluorescently labeled peptides, such as RhB, 2-Abz, and FAM, can be useful tools for the study of mechanisms of action and the identification of infections by Gram-negative and Gram-positive bacteria in vivo and in vitro.

The antibacterial activity of the peptides was further tested in *E. coli*, *S. aureus*, and *E. faecalis* clinical isolates with varying resistance profiles. In *E. coli*, peptide RWQWRWQWR retained activity (MICs 17–34 µM) regardless of the resistance phenotype. The sensitive strain 1004 exhibited the highest susceptibility (MIC = 17 µM; MBC > 135 µM), indicating a bacteriostatic effect. In contrast, resistant (129797) and MDR (301755) isolates displayed bactericidal effects (MBCs = 34 µM and 67 µM, respectively), demonstrating that the expression of ESBLs and cephalosporinases does not interfere with peptide efficacy (Table 3). These observations are consistent with previous findings for LfcinB-derived peptides containing the RWQWR motif, which exhibited antibacterial activity against clinical isolates of *E. coli* and other Gram-negative bacteria, such as *P. auruginosa* and *K. pneumoniae*, with different susceptibility profiles [49,50].

As observed in the *E. coli* ATCC 25922 strain, none of the clinical isolates showed activity with the peptide RhB-1, probably due to the large, hydrophobic structure of Rhodamine B. The peptide 1-Abz had an MIC of 62 µM and an MBC of 125 µM for all three clinical isolates while Abz-1 lost its activity against all three clinical isolates; this result indicates that the 2-Abz attached to the N-terminus of the peptide significantly interferes with the interaction of the peptide with the bacterial cell, and that the positively charged Arg at the N-terminus of the RWQWRWQWR peptide is important in the activity with these strains, since in general, it was observed that peptides RhB-1, 1-Abz, and FAM-1, with these molecules attached to the N-terminus, had an evident decrease in biological activity.

The peptide RWQWRWQWR had an 8-fold decrease in biological activity (MIC = 135 µM) in clinical isolates compared to *S. aureus* ATCC 25923 (MIC = 17 µM), indicating that *S. aureus* resistance mechanisms to antibiotics such as penicillin (shared resistance in the three clinical isolates) may probably affect the activity of the RWQWRWQWR peptide (Table 3). It has been found that increased lysyl-phosphatidylglycerol in the *S. aureus* membrane may decrease the interaction with the hydrophobic amino acids of the peptide, thus decreasing the antibacterial activity [51].

As in the *S. aureus* ATCC 25923 strain, the three clinical isolates maintained biological activity with MICs ranging from 26 to 125 µM for the peptides RhB-1, 1-Abz, and Abz-1, with the rhodaminated peptide being the one that maintained the best antibacterial activity, probably, as mentioned above, due to the hydrophobicity conferred by this fluorochrome. For its part, the FAM-1 peptide completely lost its activity in the strains evaluated (Table 3), indicating that labeling the RWQWRWQWR peptide with FAM is not a useful strategy to be used as a probe in the study of the mechanisms of action of this peptide in *S. aureus* strains.

The peptide RWQWRWQWR maintained its biological activity in resistant and sensitive clinical isolates of *E. faecalis* with respect to strain *E. faecalis* ATCC 29212. Interestingly, clinical isolate 82, resistant to streptomycin, exhibited the maximum antibacterial activity with an MIC of 67 µM (Table 3). These results contrast with what was observed by Vega et al. in 2018, where the peptide RWQWRWQWR did not show activity with clinical isolates of multidrug-resistant E. faecium [50] *E. faecium* is more resistant to antibiotics than *E. faecalis* in general, which makes it more virulent and difficult to treat [52].

The peptide RhB-1 was only active against the resistant clinical isolate 82, while it lost activity against sensitive and resistant clinical isolates 213 and 179, respectively. In contrast to what was observed in *E. coli*, conjugating 2-amino benzoic acid to the N-terminus of the peptide enhanced antibacterial activity in all three clinical isolates (MIC = 31 µM), compared to the peptide with 2-amino benzoic acid at the C-terminus (MIC = 125 µM and >125 µM), indicating that the hydrophobic characteristic that this molecule gives to the peptide in this position improves the interaction with the cell membrane. In general, the fluorinated peptides had better activity with the clinical isolates of *E. faecalis*, probably because the differences observed in the composition of the membrane phospholipids of these strains favor interactions with highly hydrophobic peptides [48].

### 3.4. Anticancer Activity

To evaluate the biological implications of fluorophore conjugation, MTT assays were performed. Both 2-Abz-labeled peptides exhibited reduced cytotoxicity compared to the unmodified palindromic peptide 1 (Table 4, Figure S14, Supplementary Materials). The peptide functionalized at the C-terminal end, 1-Abz, displayed higher cytotoxicity than at the N-terminally modified end, reducing cell viability by approximately 60% at 200 µg/mL (Table 4, Figure S14, Supplementary Materials). This suggests that the inclusion of the probe at both ends of the sequence does not induce a complete loss of activity, making it useful as a probe for studying the effect of the peptide in the cell. The inclusion of this hydrophobic portion has a significant effect on the activity of the palindromic peptide on cancer cells. These findings are consistent with previous reports in colon cancer (HT-29, CaCo-2) and prostate cancer (DU-145) cells, where peptides derived from the RWQWRWQWR sequence containing hydrophobic motifs such as Ahx and Biotin at the N-terminus decrease their cytotoxic activity [53].

Similarly, FAM-1 displayed reduced cytotoxicity in HeLa cells compared to peptide 1. FAM-1 reduced cell viability by 80% at 200 µg/mL, whereas peptide 1 achieved 50% viability under the same conditions (Table 4, Figure S14, Supplementary Materials). This decrease in cell viability may be attributed to interference from the bulky and hydrophobic FAM probe. Comparable findings have been reported for plant dehydrin-derived peptides conjugated with fluorescein, which showed no cytotoxicity in A431 squamous cells [54].

Rhodamine B conjugation in the palindromic sequence, on the other hand, maintained cytotoxicity. RhB-1 peptide exhibited an IC_50_ of 61 μM, closely matching that of the unmodified peptide 1 (IC_50_ = 52 μM) (Figure 2, Table 4). Dimeric peptides 2 and RhB-2 also demonstrated a comparable cytotoxic effect in HeLa cells, suggesting that Rhodamine B conjugation at the N-terminus does not significantly affect anticancer activity. Morphological analysis showed that cells treated with peptides 1, RhB-1, 2, and RhB-2 underwent apoptosis-like changes, including rounding, shrinkage, loss of cytoplasmic extensions, and vacuole formation, without membrane rupture, indicative of apoptosis events (Figure 3).

These results are in line with earlier reports showing that LFB, LfcinB, and short synthetic peptides derived from LfcinB induce similar morphological changes, with associated apoptotic events [27,30,31,33,53]. Confocal microscopy confirmed peptide internalization and cytoplasmic distribution. Colocalization with alpha-SMA was observed for both peptides, suggesting internalization and diffusion through the cell cytoplasm (Figure 4 and Figure S15, Table S3, Supplementary Materials). Additionally, slight parts of the HeLa cell’s nucleus seem to be correlated to the Rhodamine B peptides’ signal, suggesting a partial localization in this structure as well (Figure 4 and Figure S15, Table S3, Supplementary Materials).

The possible cellular distribution of the peptides was determined by colocalization analysis of the Rhodamine B fluorescent signal, with fluorophores used to stain the nucleus (Hoechst 33342) and cytoplasm components (SMA antibody). ImageJ 1.54f analysis software and the JaCoP plugin were used to analyze signal colocalization. The results showed that both RhB-1 and RhB-2 peptides colocalized with the SMA antibody can be internalized and distributed in the cytoplasm (Figure 4 and Figure S15, Supplementary Materials). This suggests that peptide interaction with cancer cells involves a complex process of membrane interaction without compromising membrane integrity, followed by internalization, enabling interactions with organelles and intracellular components.

Overall, the results suggest that the cytotoxic effect of RhB-1 and RhB-2 peptides is associated with their internalization into the cytoplasm and related compartments. Previous studies have reported similar behavior for bovine lactoferrin, which is internalized in mammalian cells. Apo-bLf, in particular, has been shown to enter both the cytoplasm and nucleus of HeLa cells, possibly via endocytosis [55], similar to the behavior found for RhB-1 and RhB-2. Other milk-derived synthetic peptides functionalized with fluorescein probes, such as (5-FAM)-LEQLLRLKKYKVPQ, have also demonstrated internalization in HeLa cells within one hour [56].

Although RhB-1 exists as a mixture of the open-ring and spiro-lactam forms, its fluorescent intensity remains within the range of 10^6^ units for corrected total cell fluorescence, indicating that the equilibrium between the fluorescent and non-fluorescent forms does not compromise the signal sensitivity at the IC_50_ concentration. This may be related to the high quantum yield of Rhodamine B, which can produce strong fluorescent signals even at nanomolar concentrations [26]. Consequently, Rhodamine B is a suitable probe for the development of highly sensitive fluorescence peptides based on LfcinB.

Given the comparable cytotoxic activity of RhB-1 and the palindromic peptide in MCF-7 cells, a preliminary competition assay was performed using RhB-1 and the RGD-Ahx-1 peptide, which has higher cytotoxic activity and induces apoptosis via similar mechanisms (Figure 5). When cells were treated first with RhB-1 (34 μg/mL) for 2 h, followed by treatment with RGD-Ahx-1 at increasing concentrations (0–200 μg/mL), cell viability remained constant at ~40%, while control cells treated only with RGD-Ahx-1 exhibited up to 20% viability (Figure 5B). These results suggest that RhB-1 may bind a shared receptor with RGD-Ahx-1, preventing its displacement even at high concentrations. If RhB-1 acted via nonspecific interactions, RGD-Ahx-1 would be expected to reduce viability, suggesting that the competition assay suggests a receptor-mediated mechanism for RhB-1 activity in cancer cells.

The results suggest that synthetic peptides based on the RWQWRWQWR and (RRWQWR-hF-KKLG)_2_K-Ahx sequences may interact with cancer cells through complex mechanisms involving receptor binding and internalization to exert their cytotoxic effects (Figure 5C). Studies on related peptides, such as RGD-Ahx-RWQWRWQWR, have indicated potential interactions with the extrinsic apoptotic pathway in MCF-7 cells, as well as the induction of intrinsic apoptotic mechanisms [33]. Microscopy images of HeLa cells support the idea that these peptides can access cytoplasmic and nuclear targets following internalization at their IC_50_ concentrations. These findings are consistent with the morphological alterations, induction of apoptosis, and activation of intrinsic caspases, particularly caspase-3 and caspase-7, in cancer cells treated with LfcinB, LFB, and the palindromic peptides [29,37,55]. One plausible mechanism is that the peptide first interacts with specific cellular receptors that could either activate death signals or induce its internalization to exert other functions on intracellular targets. Cell-penetrating peptides (CPPs) are typically amphiphilic [57], shared by the RWQWRWQWR sequence. The presence of Arginine residues has been shown to be critical for interaction with cell–surface glycoproteins and for mediating passive internalization in many cationic and amphipathic CPPs [58]. Therefore, the Arginine residues in these sequences may underlie the observed internalization behavior of RhB-1 and RhB-2, supporting their classification as cell-penetrating peptides.

Previous studies have demonstrated that the palindromic motif, when N-terminally functionalized with peptide amphiphilic cationic sequences, such as RRWQWR-Ahx-RWQWRWQWR and RLLRRLLR-Ahx-RWQWRWQWR, exhibited a significant and selective cytotoxic effect in cervical cancer cells, HeLa and Ca Ski. Added to this, the palindromic sequence functionalized with non-protein molecules, such as Ahx-RWQWRWQWR, caffeic acid-Ahx-RWQWRWQWR, ferulic acid-Ahx-RWQWRWQWR, and Oxinilic acid-Ahx-RWQWRWQWR, exhibited antibacterial activity against *E. coli* and *S. aureus* strains. These suggest that the incorporation of the protein and non-protein molecules in the N-terminal end did not affect the antibacterial and cytotoxic activity.

Peptides 1, 2, RhB-1, and RhB-2 showed anticancer/antibacterial activity; peptide 1 can be considered as a promising AMP/ACP because it showed activity against resistant, sensitive, and multidrug-resistant clinical isolates of Gram-positive and Gram-negative bacteria, as well as against breast, colon, cervical, oral, and prostate cancer cell lines. The therapeutic index and selectivity index for erythrocytes and fibroblasts were greater than one, indicating that this peptide is selective for bacteria and cancer cells. Toxicity tests in in vivo models, such as *Galleria mellonella*, zebrafish, and mice, show that this peptide has a medium/low toxicity, suggesting that it is a safe molecule. Optimization strategies of this peptide show that point changes in one or more positions of the palindromic sequence decreased the anticancer and antibacterial activity. However, incorporation of the molecules at the N-terminal end of the sequence maintained or increased the activity, suggesting that the palindromic sequence could be considered for anchoring protein and non-protein molecules to increase activity or use as a transporter. Incorporation of the fluorescent probes into the palindromic sequence increased activity in some bacterial strains and cancer cells, suggesting that the incorporation of non-protein molecules can be used to modulate activity toward a specific pathogen. The fact that the incorporation of complex molecules, such as Rhodamine, did not significantly affect cytotoxic activity suggests that the palindromic sequence is a versatile molecule prone to functionalization at the N-terminal end for designing and identifying preclinical assay candidates.

On the other hand, a single-residue substitution at position 26 of the dimeric peptide (RRWQWRMKKKLG)_2_K-Ahx significantly increased antibacterial and anticancer activity. Replacement of the methionine residue with hydrophobic residues, such as phenylalanine, leucine, or 1-naphthylalanine (Nal-1), to yield peptide 2, significantly increased cytotoxicity against breast cancer cells. Additionally, the antibacterial/anticancer activity of this dimeric peptide 2 is not significantly affected when Rhodamine was incorporated at the N-terminal end of both peptide chains, suggesting that point changes in sequencing and/or its functionalization at the N-terminal end are useful strategies to increase activity. These findings align with previous reports showing that the Leu by Phe substitution in the p-Acl peptide sequence (KKYKAYFKKFKCKK) increased antibacterial activity against clinical isolates and cancer cell lines [59].

### 3.5. Hemolytic Activity

Peptide 1 exhibited no detectable hemolysis and a therapeutic index > 8, consistent with its selective disruption of bacterial and cancer cell membranes [41]; this observation aligns with previous studies showing that LfcinB and its derivatives preferentially associate with acidic phospholipids, phosphatidylglycerol in bacterial membranes, and phosphatidylserine in cancer cell membranes (Figure S16 and Table S4, Supplementary Materials) [60].

N-terminal fluorolabeling of peptide 1 with fluorescein (FAM-1), Rhodamine B (RhB-1), or 2-aminobenzoic acid (Abz-1) induced a marked increase in hemolytic activity (Figure S16 and Table S4, Supplementary Materials). This rise is attributable to the enhanced hydrophobicity conferred by these probes, consistent with reports that conjugation of highly hydrophobic fluorophores to antimicrobial peptides, such as PEph3, Pepneg, VCPP2319, and CTN, similarly augments erythrocyte toxicity [26,42]. By contrast, C-terminal attachment of 2-aminobenzoic acid (1-Abz) substantially attenuated hemolysis, demonstrating that fluorophore placement exerts a critical influence on peptide and red blood cell interactions and that C-terminal labeling represents a promising strategy to preserve hemocompatibility.

## 4. Materials and Methods

### 4.1. Reagents and Materials

Rink amide MBHA resin, Fmoc-Arg(Pbf)-OH, Fmoc-Gln(Trt)-OH, Fmoc-Trp(Boc)-OH, Fmoc-Gly-OH, Fmoc-Ahx-OH, Fmoc-Lys(Boc)-OH, Fmoc-Lys(Fmoc)-OH, Fmoc-Leu-OH, 6-chloro-1-hydroxybezotriazole (6-Cl-HOBt), N,N’-Dicyclohexylcarbodiimide (DCC), and 2-(1H-Benzotriazole-1-yl)-1,1,3,3-tetramethylaminium tetrafluoroborate (TBTU) were purchased from AAPPTec (Louisville, KY, USA). Methanol (MeOH), diethyl ether, N,N-dimethylformamide (DMF), dichloromethane (DCM), absolute ethanol (EtOH), acetonitrile (ACN), formic acid (FA), isopropyl alcohol (IPA), trifluoroacetic acid (TFA), N,N-Diisopropylethylamine (DIPEA), 1,2-ethanedithiol (EDT), triisopropylsilane (TIPS), and RP-SPE Supelclean columns were obtained from Merck (Darmstadt, Germany). 2-Aminobenzoyl (2-Abz), 5(6)-Carboxyfluorescein (5(6)-FAM), Rhodamine B (RhB), 4-methylpiperidine, pyridine, triton X, potassium cyanide (KCN), phenol, ninhydrin, ethylenediaminetetraacetic acid (EDTA), RPMI-1640 culture medium, DMEM culture medium, and bovine trypsin were purchased from Sigma-Aldrich (St Louis, MO, USA). Fetal bovine serum (FBS) and Dulbecco’s Modified Eagle Medium were purchased from Gibco (Waltham, MA, USA). The HeLa and MCF-7 cell lines and *E. coli* ATCC 25922, *P. aeruginosa* ATCC 27853, *K. pneumoniae* ATCC 700603, *S. aureus* ATCC 25923, and *E. faecalis* ATCC 29212 strains were purchased from ATCC (Manassas, VA, USA). Reagents and solvents were used without further purification as they were of the purity required for each assay.

### 4.2. Synthesis of Peptides Protocol

Peptides were synthesized using Solid Phase Peptide Synthesis (SPPS) with the Fmoc/*t*Bu strategy, as previously described by Ardila et al. [30], with some modifications. Briefly, 150 mg of Rink amide resin (0.46 meq/g) was swollen by treatment with a mixture of DCM/DMF (1:1 *v*/*v*) for 2 h under constant agitation at room temperature (RT). Fmoc deprotection was performed with 5% 4-methyl-piperidine and 0.1% Triton X-100 in DMF for 15 min at RT under constant stirring (2×). The resin was washed with DMF (6 × 1 min) and DCM (3 × 1 min), and the Kaiser test was carried out.

The Fmoc amino acid pre-activation step was performed by dissolving the Fmoc-amino acid/DCC/6-Cl-HOBt (1:1:1 equiv. and five excess with respect to the resin substitution) in 2 mL of DMF, and the mixture was stirred for 15 min at RT. The reaction mixture was mixed with the resin or resin–peptide, in which previously the Fmoc group was removed, and the reaction was gently stirred for 12 h at RT. The solution was filtrated, and the resin or resin–peptide was washed with DMF (3 × 1 min), IPA (1 × 1 min), and DCM (3 × 1 min). The Kaiser test was carried out, and if the test was positive, the coupling reaction was repeated until we obtained a negative test. For the synthesis of dimeric peptides, the epsilon and alpha amine groups of Fmoc-Lys(Fmoc)-OH were employed to build two peptide chains. The pre-activation amino acid was performed using Fmoc-amino acid/DCC/6-Cl-HOBt (1:1:1 equiv. and 10 excess with respect to the resin substation).

Incorporation of the fluorescent probe (Fmoc-2-amino benzoic acid (Fmoc-2-Abz), 5(6)-carboxyfluorescein (5(6)-FAM), or Rhodamine B (RhB)) into the peptide sequence in SPPS-Fmoc/tBu was carried out by amide bond formation. The selected probes contain a carboxylic acid group that reacts with the alpha-amino group of the resin, or the terminal amino acid anchored to the resin. The coupling reactions were carried out using TBTU and DIPEA as activators to form a reactive ester, which subsequently reacted with H_2_N-RWQWRWQWR-resine (via acyl nucleophilic substitution), yielding the following labeled peptides: 5(6)-FAM-RWQWRWQWR (FAM-1), RhB-RWQWRWQWR (RhB-1), 2-Abz-RWQWRWQWR (Abz-1), or RWQWRWQWR-2-Abz (1-Abz). Incorporation of the 2-Abz at the C-terminal end of the palindromic sequence was carried out by attaching Fmoc-2-Abz to the deprotected resin to form Fmoc-2-Abz-resin, and then the other Fmoc-amino acids were incorporated sequentially. The fluorescent molecule mixed with TBTU–DIPEA (1:1:3 equiv. and three excess with respect to resin substitution and six excess for the dimeric peptide) was prepared. The reaction mixture was added to the resin–peptide and the reaction was left to proceed for 1 h at RT. Then, the mixture reaction was discarded by filtration, and the resin–peptide was washed with DMF (10 × 1 min) and DCM (6 × 1 min), and the Kaiser test was carried out. Once the fluorescent probe was attached to the chain, the resin–peptide was dried, weighted, and treated (1:20 *w*/*v*) with a solution of TFA/Water/TIPS/EDT (92.5/2.5/2.5/2.5 *v*/*v*) for 6 h at RT and shaking. The reaction mixture was filtered, the solution was treated with ether ethylic to precipitate the peptide, and it was washed with ether ethylic five times. The Kaiser test was carried out; we dried a small portion (3–5 mg) of resin and transferred it to an Eppendorf tube and treated it with 300 µL of a mixture of solution A–solution B (1:2 *v*/*v*), heating at 105 °C for 5 min. Solution 1: 40 g phenol/10 mL EtOH, and Solution 2: 1 mL of potassium cyanide (65 mg/100 mL of water) was diluted with 50 mL of pyridine. Solution A: a mixture of Solutions 1 and 2 (1:1 *v*/*v*). Solution B: 1.25 g of Ninhydrin/25 mL EtOH.

### 4.3. Peptide Purification by RP-SPE

Peptides were purified using reversed-phase solid-phase extraction (RP-SPE) as described by Insuasty et al. [29]. Briefly, the RP-SPE columns (Supelclean LC-18 SPE Tube, 2 g) were conditioned according to the manufacturer’s recommendations. Crude peptides were dissolved in water (1 mg/mL), analyzed by RP-HPLC, and their elution %B (TFA 0.05% in ACN) was determined to design a stepwise gradient. Solutions with increasing %B were prepared and passed through the column. Fractions (12 mL) were collected and analyzed by RP-HPLC; those containing high-purity peptides were pooled, ACN was removed by evaporation, and the peptides were lyophilized. Final purity was confirmed by RP-HPLC.

### 4.4. Characterization by RP-HPLC and High-Resolution LC-MS

Peptides (10 µL, 1 mg/mL in 0.05% TFA in water) were analyzed using a Hitachi Primaide-DAD 1110 system and a Chromolith Performance RP-18 endcapped 100-4.6 column. Solvent B was ACN with 0.05% TFA. The elution gradient was 20/20/50/100/100/20/20% B at 0/1/9/9.1/12/12.1/17 min, with a flow rate of 1 mL/min at RT. Wavelengths were scanned from 200 to 400 nm; analysis was performed at 210 nm.

For LC-MS, peptides (2 µL, 0.01 mg/mL) were analyzed using a Bruker Impact II LC-QTOF MS system (ESI-positive mode). Separation was performed on an Intensity Solo 2 C-18 column (100 × 2.1 mm, 2 µm). Solvent A was 0.1% formic acid in water; solvent B was 0.1% formic acid in ACN. The elution gradient was 5/5/95/95/5/5% B at 0/1/11/13/13.1/ 15 min at a flow rate of 0.25 mL/min. ESI source settings: end plate offset 400 V, capillary 4000 V, nebulizer 26.1 psi, dry gas nitrogen 8,0 L/min, and dry temperature 220 °C. Mass spectra were acquired in Auto MS/MS mode, range 50–1300 m/z, rate 6 Hz.

### 4.5. Bacterial Strains and Culture Conditions

The analysis of synthesized peptides in ATCC reference strains and susceptible, resistant, and multidrug-resistant (MDR) clinical isolates was performed. The clinical isolates were obtained from the National Cancer Institute collection and were previously identified and antibiogrammed using the VITEK system. Additionally, the strains were characterized by MALDI-TOF. The strains were re-primed onto trypticase soy agar and subsequently preserved in Brain–Heart Infusion (BHI) broth containing 20% glycerol. For the MIC and MBC assays, the strains were reactivated and cultured in BHI until they reached their exponential growth phase.

### 4.6. Minimum Inhibitory Concentration

The minimum inhibitory concentration (MIC) of the peptides was determined by the broth microdilution technique, according to the Clinical and Laboratory Standards Institute (CLSI) guidelines, as previously described [49]. Briefly, bacterial strains were incubated in BHI broth at 37 °C until they reached the exponential growth phase, and inocula were adjusted to 5 × 10^6^ CFU/mL using calibration curves. Serial dilutions of the peptides to be evaluated in 96-well plates were made in 90 µL of Mueller Hinton broth at concentrations ranging from 200 to 6.25 µg/mL, and 10 µL of the inoculum adjusted to 5 × 106 CFU/mL was added. Finally, the plates were incubated at 37 °C for 24 h, and the absorbance at 620 nm was read. As a negative control, CMH, distilled water, and 10 µL of inoculum were used; for the positive control, vancomycin was used for Gram-positive strains and ciprofloxacin for Gram-negative strains at the MICs dictated by the CLSI; as a technical control, CMH with peptone water was used, and for the growth control, CMH was used with 10 µL of inoculum.

### 4.7. Minimum Bactericidal Concentration

To determine the minimum bactericidal concentration (MBC), the 96-well plates where the MIC was determined were used as a starting point. From these, an aliquot of the controls and those wells where no apparent bacterial growth was observed were spotted onto trypticase soy agar plates. The plates were incubated at 37 °C for 24 h, and the reading was taken, identifying the lowest concentration of the peptide where there was no bacterial growth as the MBC.

### 4.8. Fluorescence Confocal Microscopy Imaging

HeLa cells (>70% confluence) were seeded on sterile round coverslips in 24-well plates with DMEM + 5% FBS and incubated at 37 °C, 5% CO_2_ for 24 h. After adhesion, the medium was replaced, and cells were treated with fluorescent peptides at their IC_50_ for 2 h. Then, cells were washed with PBS under constant stirring (3 × 10 min). Cellular fixation was performed using a solution containing 2% paraformaldehyde and 1% sucrose 100 mM in PBS for 30 min, followed by PBS washes (2 × 10 min). Permeabilization was carried out using a solution of 0.1% Triton X-100 and 20 mM glycine in PBS for 10 min, followed by two additional PBS washes. Blocking was performed with 1% BSA and 20 mM glycine in PBS for 10 min. Subsequently, cells were incubated with an anti-alpha-smooth muscle actin antibody (SMAC) labeled with Alexa Fluor 488 for 90 min in the dark. After incubation, cells were washed with PBS for 10 min. To stain the nuclei, 25 µL of Hoechst 3342 solution (1:50 dilution) was added and incubated for 7 min, followed by two PBS washes (2 × 10 min). The coverslips were dried by capillarity. For the fluorescence lecture, 5 µL of Fluoromount mounting medium was applied to the slide, and the coverslip containing the cells was inverted onto the medium. Slides were allowed to be dry-protected from light, and cells were visualized using a Leica DMi8 fluorescence confocal microscope. LAS X 3.10.0 and Fiji ImageJ 1.54f software were used for image analysis.

### 4.9. Competition Assay in MCF-7 Cells

A preliminary competition assay was conducted to explore potential receptor-mediated interactions between peptides. MCF-7 cells were first treated with the fluorinated peptide RhB-1 at its IC_50_ concentration (34 μg/mL) for 2 h at 37 °C. After incubation, the culture medium was removed, and a fresh medium containing the RGD-Ahx-1 peptide was added at increasing concentrations (0–200 μg/mL). The cells were incubated for an additional 2 h under the same conditions. Cell viability was assessed using the MTT assay. As a control, a parallel set of cells was treated only with the RGD-Ahx-1 peptide for 4 h under identical conditions. All treatments were performed in triplicate.

### 4.10. Hemolysis Assay

Whole blood was collected into EDTA tubes and centrifuged at 2500 rpm for 10 min. The plasma and buffy coat were removed, and the erythrocytes were washed three times with 0.9% saline to yield a 4% cell suspension. In 96-well plates, 100 µL of peptide dilutions (6.25–200 µg/mL) were prepared in triplicate, and 100 µL of the erythrocyte suspension was added (final hematocrit 2%). Plates were incubated at 37 °C for 2 h, and then centrifuged again at 2500 rpm for 10 min. A 100 µL aliquot of the supernatant was transferred to a fresh plate, and absorbance was measured at 450 nm. Saline and 1× Triton X-100 served as the negative and positive controls, respectively. Hemolysis (%) was calculated as follows:

$$\%hemolysis=\frac{treatment\;absorbance-saline\;absorbance}{1\times triton\;absorbance-saline\;absorbance}$$

## 5. Conclusions

The fluorolabeled peptides’ obtention is complex and often leads to the formation of unwanted by-products and mixed isomers. The efficiency depends mainly on the sequence, the type of fluorescent probe, and the binding site of both the probe and the sequence. The fluorolabeled peptides showed high antibacterial activity against sensitive, resistant, and multidrug-resistant clinical isolates of E. coli, *S. aureus*, and *E. faecalis*. RhB-1 and RhB-2 peptides were internalized in HeLa cells and localized to both the cytoplasm and nucleus, suggesting their action may involve interactions with intracellular targets, although membrane receptors may also play a role. These findings suggest that fluorolabeling of antimicrobial peptides (AMPs) could be a promising strategy for the development of novel antibacterial and anticancer agents. Overall, these findings underscore the potential of fluorolabeled AMPs as molecular probes for elucidating mechanisms of action and for diagnosing bacterial infections.

## Figures and Tables

**Table 1:** Characterization of fluorolabeled peptides and unlabeled peptides 1 and 2, including the probe position within each sequence, the retention time (t_R_), and the experimentally determined monoisotopic mass of each peptide.

Code	Sequence	HPLC		LC-MS Monoisotopic Mass	
		t_R_ (min)	Purity (%)	Expected	Observed
1	RWQWRWQWR	6.8	93.1	1485.7600	1485.7596
1-Abz	RWQWRWQWR-(2-Abz)	7.5	88.4	1604.8000	1604.7980
Abz-1	(2-Abz)-RWQWRWQWR	7.3	89.7	1604.8000	1604.7992
FAM-1	FAM-RWQWRWQWR	8.2	94.2	1843.8100	1843.8112
RhB-1	RhB-RWQWRWQWR	P1: 9.6	28.5	1910.9900	P1: 1910.9830 P2: 1910.9830
		P2: 9.9	65.4		
		Overall purity: 93.4			
2	(RRWQWR-hF-KKLG)_2_K-Ahx	6.6	97.2	3370.0100	3370.0051
RhB-2	(RhB-RRWQWR-hF-KKLG)_2_K-Ahx	9.9	98.4	4220.4500	4220.4444

**Table 2:** Antibacterial activity against reference strains of fluorolabeled peptides. Minimal inhibitory concentration (MIC) and minimal bactericidal concentration (MBC) are expressed in micromolars (µM). The symbol > indicates that the MIC or MBC exceeded the highest concentration tested.

Code	Sequence	*E. coli*	*P. aeruginosa*	*K. pneumoniae*	*S. aureus*	*E. faecalis*
		ATCC 25922	ATCC 27853	ATCC 700603	ATCC 25923	ATCC 29212
1	RWQWRWQWR	17/34	67/>135	67/135	17/34	135/>135
RhB-1	RhB-RWQWRWQWR	>105/>105	>105/>105	>105/>105	26/52	52/52
1-Abz	RWQWRWQWR-Abz	62/62	125/>125	125/125	31/52	125/>125
Abz-1	2-Abz-RWQWRWQWR	62/125	>125/>125	>125/>125	16/31	62/62
FAM-1	FAM-RWQWRWQWR	108/>108	>108/>108	>108/>108	108/>108	54/>108

**Table 3:** Antibacterial activity of peptides in clinical isolates of *E. coli*, *S. aureus*, *and E. faecalis*. The minimal inhibitory concentration (MIC)/minimal bactericidal concentration (MBC) are expressed in µM concentration. The symbol > indicates that it was not possible to determine the MIC or MBC at the evaluated peptide concentrations.

Code	Sequence	Clinical Isolates								
		*E. coli*			*S. aureus*			*E. faecalis*		
		1004	129797	301755	109095	11719	124653	213	82	179
1	RWQWRWQWR	17/>135	34/34	34/67	135/135	135/>135	135/>135	135/135	67/67	135/135
RhB-1	RhB-RWQWRWQWR	>105/>105	>105/>105	>105/>105	26/105	52/105	105/105	>105/>105	26/105	>105/>105
1-Abz	RWQWRWQWR-Abz	62/125	62/125	62/125	125/125	125/125	125/>125	>125/>125	>125/>125	>125/>125
Abz-1	Abz-RWQWRWQWR	>125/>125	>125/>125	>125/>125	125/>125	125/>125	125/>125	31/62	31/62	31/62
FAM-1	FAM-RWQWRWQWR	108/>108	>108/>108	108/>108	>108/>108	>108/>108	>108/>108	108/>108	54/>108	>108/>108

**Table 4:** Cytotoxic activity of conjugated peptides and related control peptides in HeLa cells.

Code	Sequence	Cytotoxic Activity (IC_50_)	
		μg/mL	μM
1	RWQWRWQWR	77	52
1-Abz	RWQWRWQWR-(2-Abz)	>200	>125
Abz-1	(2-Abz)-RWQWRWQWR	140	87
FAM-1	FAM-RWQWRWQWR	>200	>108
RhB-1	RhB-RWQWRWQWR	117	61
2	(RRWQWR-hF-KKLG)_2_K-Ahx	109	32
RhB-2	(RhB-RRWQWR-hF-KKLG)_2_K-Ahx	151	35

## Data Availability

All data generated or analyzed during this study are included in this published article and its Supplementary Materials.

## Supplementary Materials

The following supporting information can be downloaded at https://www.mdpi.com/article/10.3390/antibiotics14080793/s1, Figure S1: Characterization of peptide 1-Abz (RWQWRWQWR-(2-Abz)) by RP-HPLC and ESI-QTOF. Figure S2: Characterization of peptide Abz-1 ((2-Abz)-RWQWRWQWR) by RP-HPLC and mass spectrometry. Figure S3: Analytical profile of FAM-1 peptide (5(6)-FAM-RWQWRWQWR). Figure S4: LC-MS analysis of RhB-1 peptide showing isomeric species. Figure S5: UV chromatogram (210 nm) of RhB-1 showing open-ring and spiro-lactam forms. Figure S6: Visible absorbance chromatogram (562 nm) of RhB-1 indicating open-ring form. Figure S7: Mass spectrum of RhB-1 (t_R_ = 9.6 min) with isotopic distribution. Figure S8: Mass spectrum of RhB-1 (t_R_ = 9.9 min) showing multiple charge states. Figure S9: UV chromatogram (210 nm) of RhB-2 peptide. Figure S10: Visible chromatogram (562 nm) of RhB-2 peptide. Figure S11: ESI-QTOF spectrum and isotopic profile of RhB-2 peptide. Figure S12A. Chromatogram of RhB-1 analyzed with the method 0/1/11/11.1/13/13.1/15 min—20/20/50/100/100/20/20 B%. Equilibrium in time of spiro-lactam (P1) and open-ring forms (P2). Figure S12B. UV–Vis spectra of the peptide RhB-1 recorded at pH 3 (purple), pH 7 (green), and pH 9 (blue) in the range of 200–700 nm, measured using a Thermo Scientific™ GENESYS™ 150 UV–Visible spectrophotometer. Table S1: Resistance profile of clinical isolates of *E. coli*, *S. aureus*, and *E. faecalis*. Table S2: Spectral characterization and peak purity of RhB-1 and RhB-2 peptides. Figure S13: DAD topogram and UV–VIS spectra of RhB-1 highlighting open-ring absorbance. Figure S14: Cell viability assays of labeled peptides in HeLa cells. Figure S15: Confocal microscopy images showing RhB-2 localization in HeLa cells. Table S3: Quantitative colocalization analysis using ImageJ JaCoP plugin. Figure S16: Hemolytic activity of peptides. Table S4: Hemolytic activity, selective index, and therapeutic index of evaluated peptides.
