# Supplementary material for: Fluorescent Peptides Internalize HeLa Cells and Kill Multidrug-Resistant Clinical Bacterial Isolates
Source: Antibiotics (Basel). 2025 Aug 4;14(8):793. doi: 10.3390/antibiotics14080793 (PMC12383145; doi:10.3390/antibiotics14080793)
Supplement: Supplementary file 1 [file antibiotics-14-00793-s001.zip › antibiotics-3748818-supplementary.pdf]

antibiotics

Article

Fluorescent Peptides Internalize HeLa Cells and Kill Multi-Drug-Resistant Clinical Bacterial Isolates

Daniel Castellar-Almonacid [1], Kelin Johana Cuero-Amu [2], Jose David Mendoza-Mendoza [2], Natalia Ardila-Chantré [1], Fernando José Chavez-Salazar [3], Andrea Carolina Barragán-Cárdenas [4], Claudia Parra-Giraldo [6], Jhon Erick Rivera-Monroy [5], Zuly Jenny Rivera-Monroy [3], Javier García-Castañeda [1,*] and Ricardo Fierro-Medina [3]

1 Departamento de Farmacia, Facultad de Ciencias, Universidad Nacional de Colombia-Sede Bogotá, Carrera 45 No 26-85, Building 451, Bogotá D.C. 111321, Colombia; dcastellara@unal.edu.co (D.C.-A.); nardilac@unal.edu.co (N.A.-C.)
2 Instituto de Biotecnología, Facultad de Ciencias, Universidad Nacional de Colombia-Sede Bogotá, Carrera 45 No 26-85, Building 451, Bogotá D.C. 111321, Colombia; kcueroa@unal.edu.co (K.J.C.-A.); jomendozam@unal.edu.co (J.D.M.-M.)
3 Departamento de Química, Facultad de Ciencias, Universidad Nacional de Colombia-Sede Bogotá, Carrera 45 No 26-85, Building 451, Bogotá D.C. 111321, Colombia; fchavezs@unal.edu.co (F.J.C.-S.); zjriveram@unal.edu.co (Z.J.R.-M.); rfierrom@unal.edu.co (R.F.-M.)
4 Bacteriología y Laboratorio Clínico, Facultad de Ciencias de la Salud, Universidad Colegio Mayor de Cundinamarca, Bogotá D.C. 110311, Colombia; abarraganc@unal.edu.co
5 Laboratorio Instrumental de Alta Complejidad, Universidad de La Salle, Carrera 5 No. 59A-44, Bogotá D.C. 110231, Colombia; jhrivera@lasalle.edu.co
6 Biomedical Sciences Faculty, Universidad Europea, Madrid, Spain 28670; claudia.parra@universidadeuropea.es
* Correspondence: jaegarciaca@unal.edu.co

Academic Editor: Gill Diamond

Received: 25 June 2025
Revised: 22 July 2025
Accepted: 31 July 2025
Published: 4 August 2025

Citation: Castellar-Almonacid, D.; Cuero-Amu, K.J.; Mendoza-Mendoza, J.D.; Ardila-Chantre, N.; Chavez-Salazar, F.J.; Barragán-Cárdenas, A.C.; Rivera-Monroy, J.E.; Parra-Giraldo, C.; Rivera-Monroy, Z.J.; García-Castañeda, J.; et al. Fluorescent Peptides Internalize HeLa Cells and Kill Multidrug-Resistant Clinical Bacterial Isolates. *Antibiotics* **2025**, *14*, 793. https://doi.org/10.3390/antibiotics14080793

Copyright: © 2025 by the author. Licensee MDPI, Basel, Switzerland. This article is an open access article distributed under the terms and conditions of the Creative Commons Attribution (CC BY) license (https://creativecommons.org/licenses/by/4.0/).

The Supporting Information includes the following:

- Figure S1: Characterization of peptide 1-Abz (RWQWRWQWR-(2-Abz)) by RP-HPLC and ESI-QTOF.
- Figure S2: Characterization of peptide Abz-1 ((2-Abz)-RWQWRWQWR) by RP-HPLC and mass spectrometry.
- Figure S3: Analytical profile of FAM-1 peptide (5(6)-FAM-RWQWRWQWR).
- Figure S4: LC-MS analysis of RhB-1 peptide showing isomeric species.
- Figure S5: UV chromatogram (210 nm) of RhB-1 showing open-ring and spiro-lactam forms.
- Figure S6: Visible absorbance chromatogram (562 nm) of RhB-1 indicating open-ring form.
- Figure S7: Mass spectrum of RhB-1 (tR = 9.6 min) with isotopic distribution.
- Figure S8: Mass spectrum of RhB-1 (tR = 9.9 min) showing multiple charge states.
- Figure S9: UV chromatogram (210 nm) of RhB-2 peptide.
- Figure S10: Visible chromatogram (562 nm) of RhB-2 peptide.
- Figure S11: ESI-QTOF spectrum and isotopic profile of RhB-2 peptide.
- Figure S12A. Chromatogram of RhB-1 analyzed with the method 0/1/11/11.1/13/13.1/15 min – 20/20/50/100/100/20/20 B%. Equilibrium in time of spiro-lactam (P1) and open-ring forms (P2).
- Figure S12B. UV-Vis spectra of the peptide RhB-1 recorded at pH 3 (purple), pH 7 (green), and pH 9 (blue) in the range of 200–700 nm, measured using a Thermo Scientific™ GENESYS™ 150 UV-Visible spectrophotometer.
- Table S1: Resistance profile of clinical isolates of E. coli, S. aureus and E. faecalis.
- Table S2: Spectral characterization and peak purity of RhB-1 and RhB-2 peptides.
- Figure S13: DAD topogram and UV-VIS spectra of RhB-1 highlighting open-ring absorbance.
- Figure S14: Cell viability assays of labeled peptides in HeLa cells.
- Figure S15: Confocal microscopy images showing RhB-2 localization in HeLa cells.
- Table S3: Quantitative colocalization analysis using ImageJ JaCoP plugin
- Figure S16: Hemolytic activity of peptides.
- Table S4: Hemolytic activity, selective index and therapeutic index of evaluated peptides

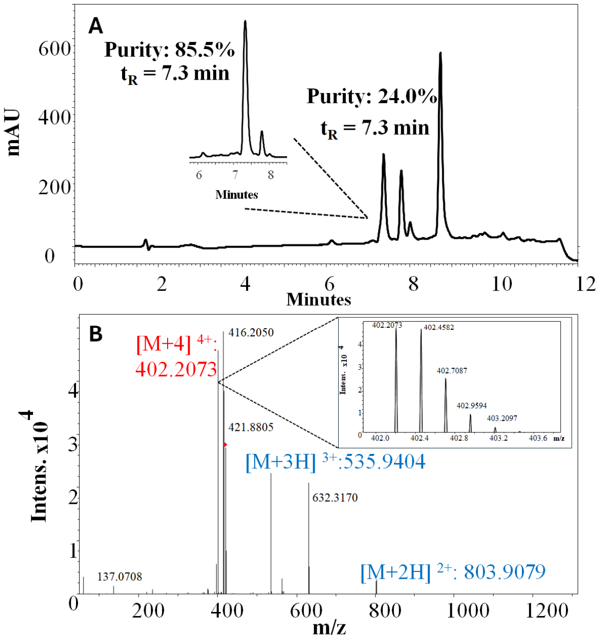

Figure S1. Characterization of peptide 1-Abz: RWQWRWQWR-(2-Abz). A) Chromatographic profile of crude peptide (purity 24.0 %) and purified peptide (purity 85.5 %). B) Mass spectrum by ESI-QTOF. The base peak corresponding to the [M+4H]4+ species (red) and the inset shows the broadening of the isotopic distribution.

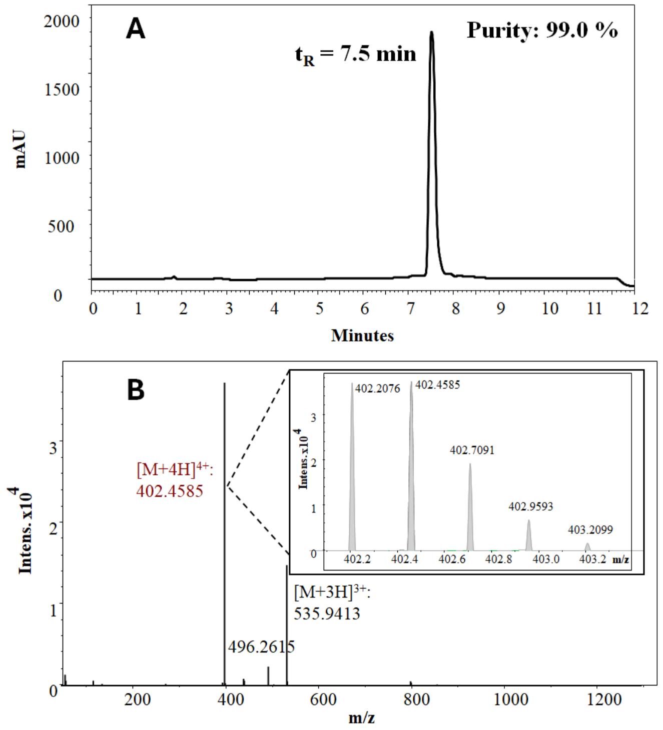

Figura S2. A) Characterization of peptide Abz-1: (2-Abz)-RWQWRWQWR. A) Chromatographic profile of purified peptide. B) Mass spectrum by ESI-QTOF. The base peak corresponding to the [M+4H]$^{4+}$ species (red) and the inset shows the broadening of the isotopic distribution.

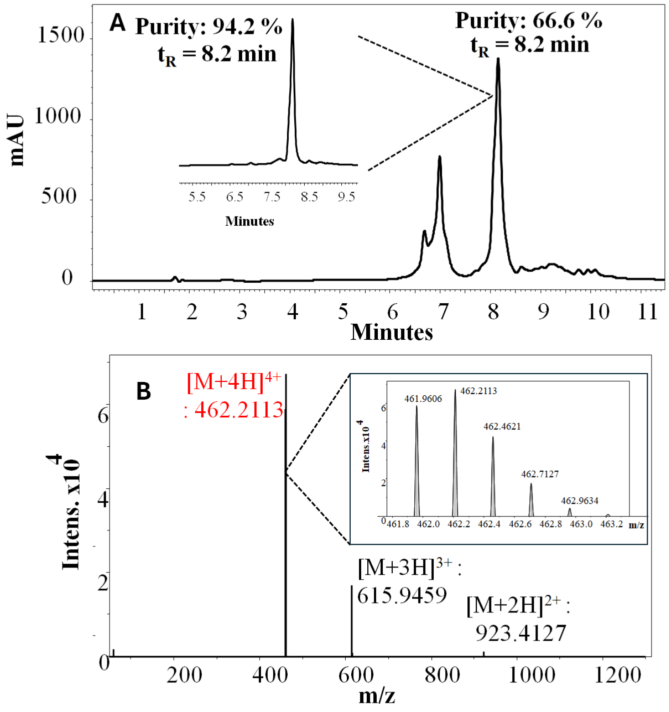

Figure S3. Characterization of the FAM-1 peptide: 5(6)-FAM-RWQWRWQWR. A) Chromatographic profile of the crude peptide (purity 66.6%) and purified peptide (purity 94.2%). B) Mass spectrum by ESI-QTOF. The base peak corresponding to the [M+4H]$^{4+}$ species is highlighted (red) and the isotopic distribution is shown in the inset.

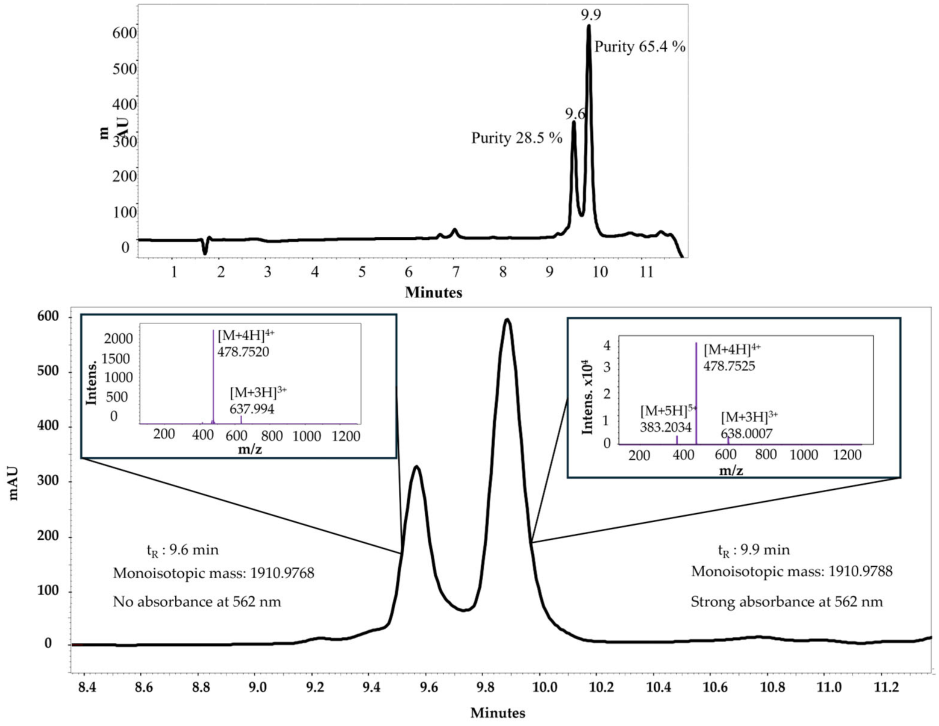

Figure S4. Characterization of the RhB-1 peptide: RhB-RWQWRWQWR by RP-HPLC and LC-MS. A) Chromatographic profile of the pure peptide showing the presence of two species with t_R= 9.6 min and t_R= 9.9 min. B) Magnification of the chromatogram shows the two species and their mass spectra by ESI-QTOF (inset), confirming that they have the same monoisotopic mass.

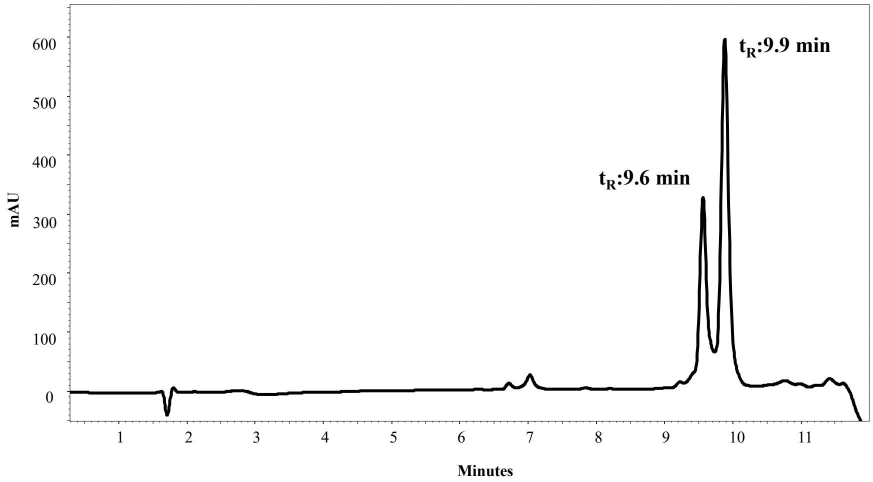

Figure S5. Chromatographic profile of RhB-1: RhB-RWQWRWQWR at 210 nm. The chromatogram shows two main peaks corresponding to the spirolactam form (t_R= 9.6 min) and the open ring form (t_R= 9.9 min).

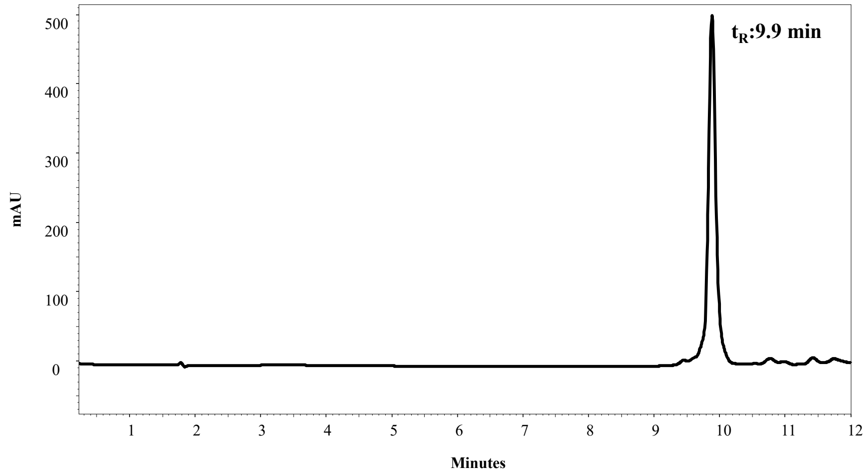

Figure S6. Chromatographic profile of RhB-1: RhB-RWQWRWQWR at 562 nm. The chromatogram shows a main peak corresponding to the open ring form (t_R= 9.9 min).

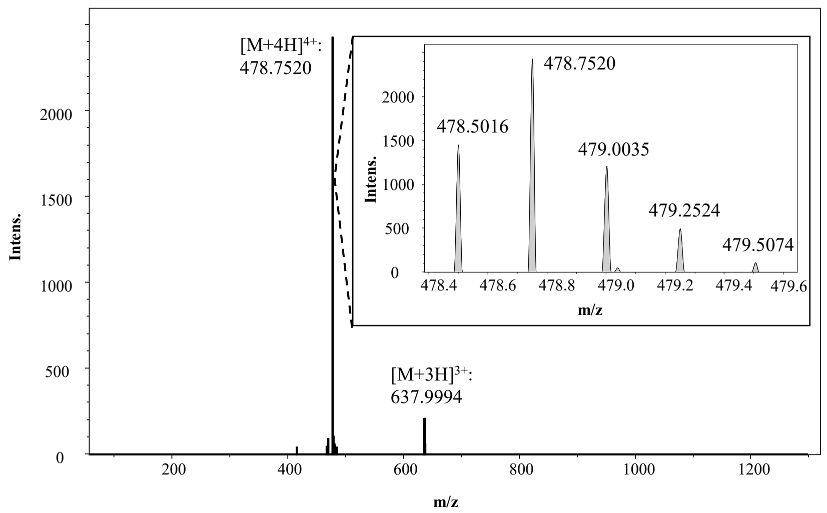

Figure S7. ESI-QTOF mass spectra and isotopic distribution pattern of RhB-1: RhB-RWQWRWQWR. The mass spectrum of the peak with tR= 9.6 min shows two multicharged [M+3H]3+ and [M+4H]4+ species. The isotopic distribution of the base peak is shown in the inset.

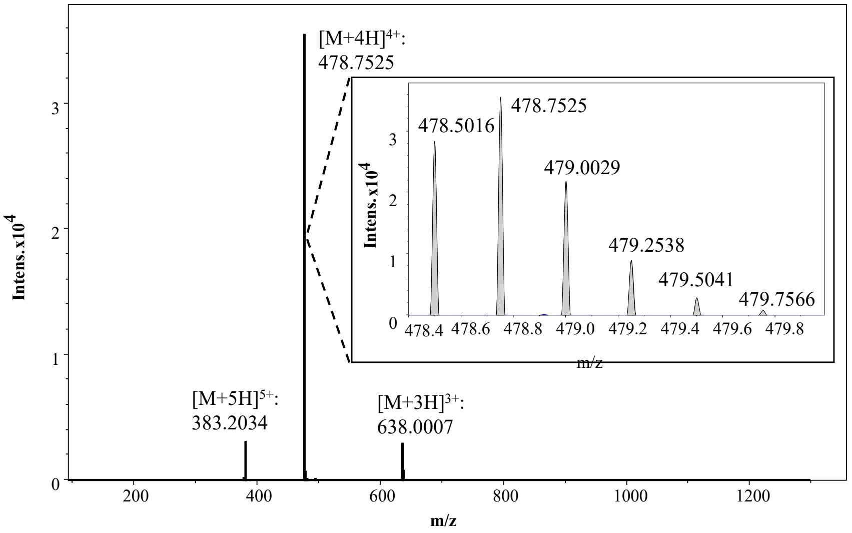

Figure S8. ESI-QTOF mass spectra and isotopic distribution pattern of RhB-1: RhB-RWQWRWQWR. The mass spectrum of the peak with tR= 9.9 min shows two multicharged $[M+3H]^{3+}$, $[M+4H]^{4+}$ and $[M+5H]^{5+}$ species. The isotopic distribution of the base peak is shown in the inset.

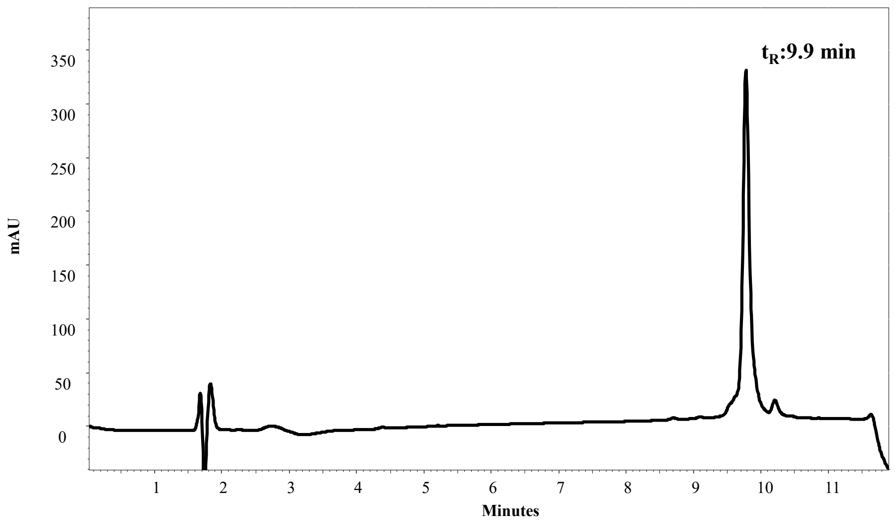

Figure S9. Chromatographic profile of RhB-2: (RhB-RRWQWR-hF-KKLG)2K-Ahx at 210 nm. The chromatogram shows a main peak corresponding to the open ring form (t_R= 9.9 min).

.

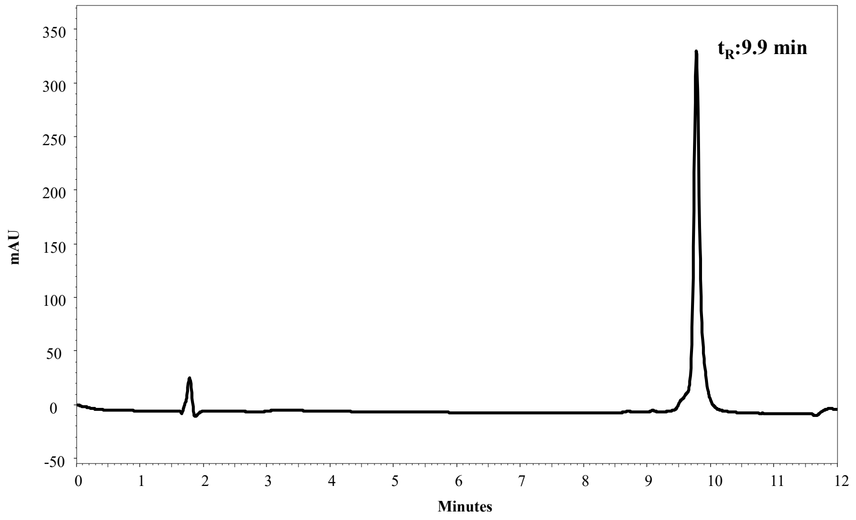

Figure S10. Chromatographic profile of RhB-2: (RhB-RRWQWR-hF-KKLG)2K-Ahx at 562 nm. The chromatogram shows a main peak corresponding to the open ring form (t_R= 9.9 min).

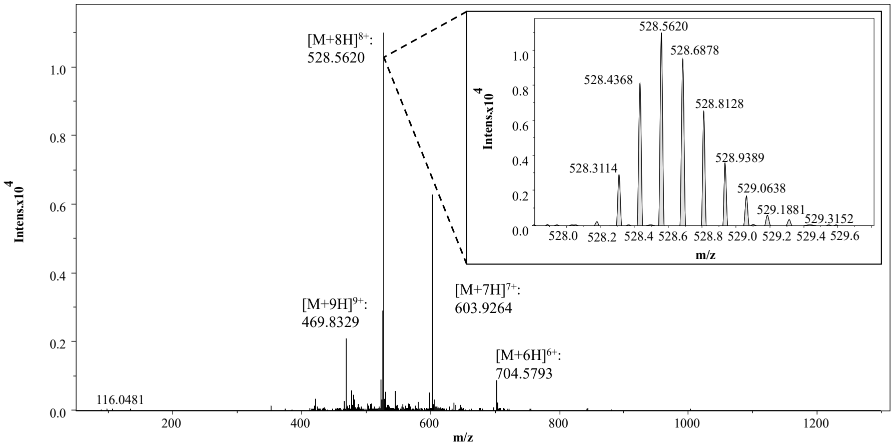

Figure S11. ESI-QTOF mass spectra and isotopic distribution pattern of RhB-2: (RhB-RRWQWR-hF-KKLG)$_2$K-Ahx. The mass spectrum of the peak with tR= 9.9 min shows two multicharged $[M+7H]^{7+}$, $[M+8H]^{8+}$ and $[M+9H]^{9+}$ species. The isotopic distribution of the base peak is shown in the inset.

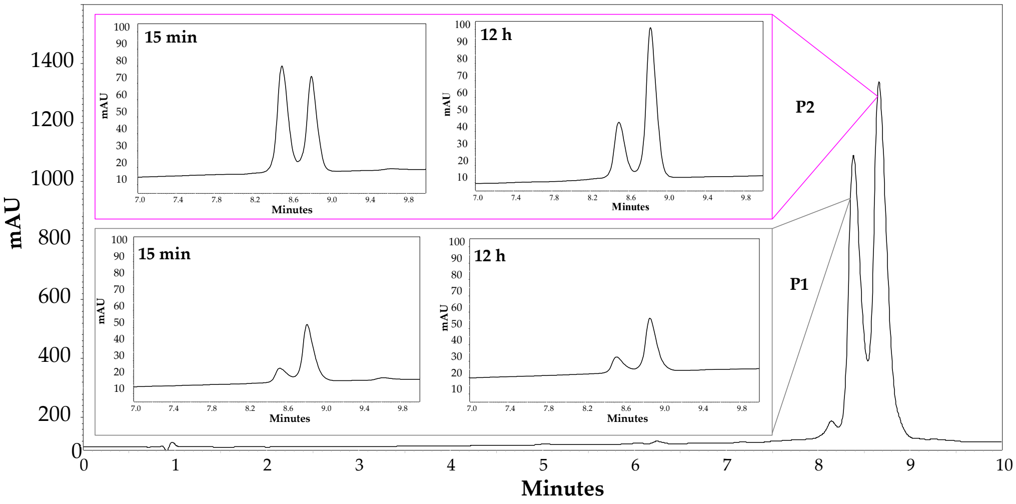

Figure S12A. Chromatogram of RhB-1 analyzed with the method 0/1/11/11.1/13/13.1/15 min – 20/20/50/100/100/20/20 B%. Equilibrium in time of spiro-lactam (P1) and open-ring forms (P2).

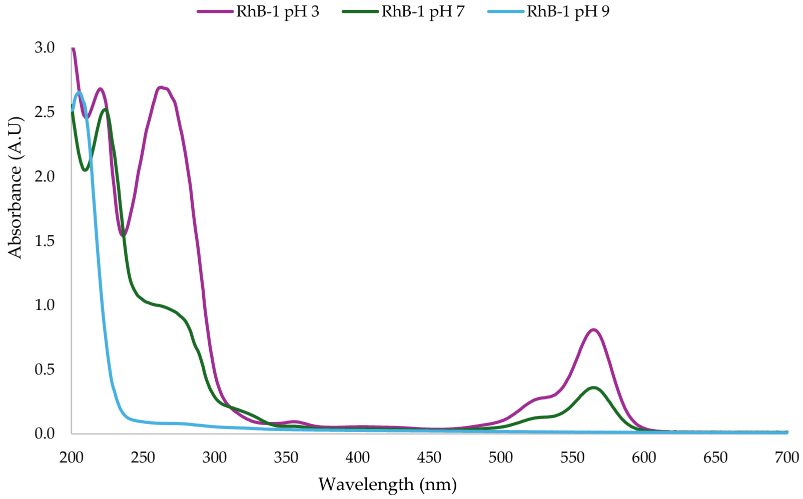

Figure S12B. UV-Vis spectra of the peptide RhB-1 recorded at pH 3 (purple), pH 7 (green), and pH 9 (blue) in the range of 200–700 nm, measured using a Thermo Scientific™ GENESYS™ 150 UV-Visible spectrophotometer.

Table S1. Resistance profile of clinical isolates of *E. coli*, *S. aureus* and *E. faecalis.*

Bacterium	Isolation Code	Antibiotic resistance	Classification
E. coli	1004	None	Sensitive
	129797	AM, CEF, SAM	Resistant
	301755	AM, CAX, CAZ, CIP, CPE, GEN, NIT, NOR, SAM, SXT	Multidrug resistant
S. aureus	109095	P	Resistant
	117719	P, TET	Resistant
	124653	P, TET, ERY	Multidrug resistant
E. faecalis	213	None	Sensitive
	82	STR	Resistant
	179	GEN	Resistant

P: Penicillin; CIP: Ciprofloxacin; CAZ: Ceftazidime; TET: Tetracycline; AM: Ampicillin; CPE: Cefepime; ERY: Erythromycin; CEF: Cephalothin; CAX: Ceftriaxone; NIT: Nitrofurantoin; NOR: Norfloxacin, GEN: Gentamicin; SAM: Ampicillin/Sulbactam; SXT: Trimethoprim/sulfamethoxazole; STR: Streptomycin

Table S2. Spectral Characterization of peptides RhB-1 and RhB-2. Peak purity percentage in DAD analysis for labelled peptides. Purity threshold was defined at 0.9700 (97 %).

Peptide	Retention time (min)	% Peak purity (200-700 nm)
RhB-1	9,6	100.0
	9,9	98.9
RhB-2	9,9	95.0

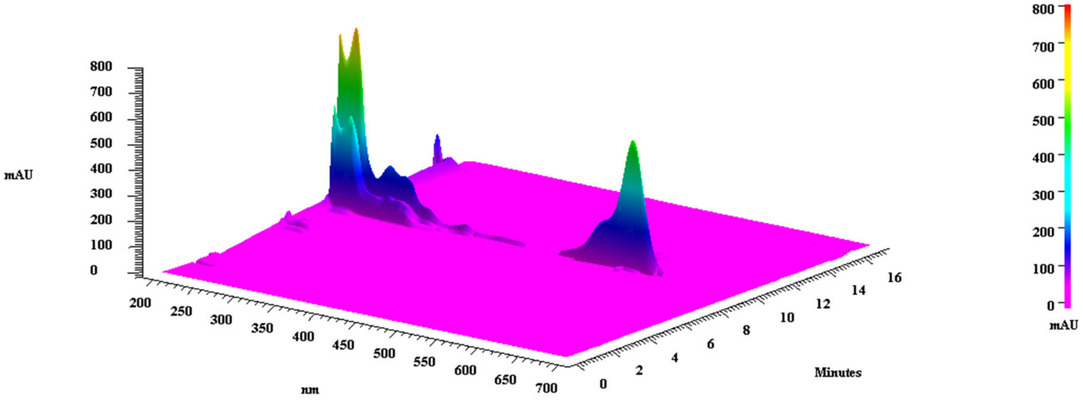

Figure S13. DAD topogram of peptide RhB-1, depicting two major UV-VIS spectra. One of them corresponds to the fluorescent open-ring form, characterised by a strong absorbance peak at 562 nm.

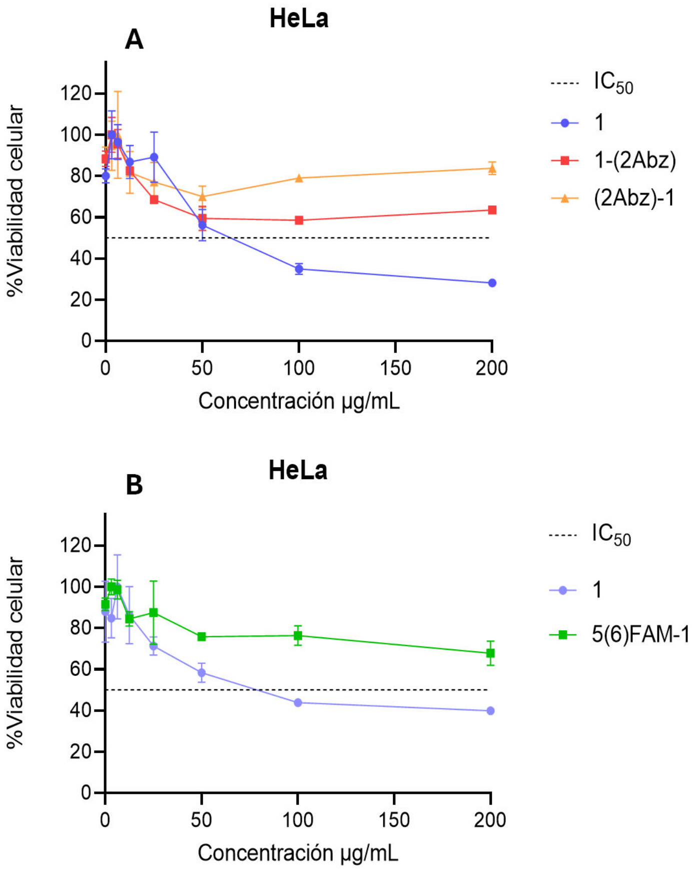

Figure S14. Cell viability plots in HeLa cells of the conjugated peptides 1-Abz, Abz-1 and FAM-1 compared to the not-conjugated parent peptide 1. Experiments were conducted in triplicate (n=3). Data are showed as mean ±S.D. Two-way ANOVA and Sidak's multiple comparison test were performed ($p<0.05$). No statistically significant differences were observed between RhB-1 and peptide 1 across the tested concentration range. RhB-2 showed statistically significant differences compared to peptide 2 at 100 and 200 µg/mL.

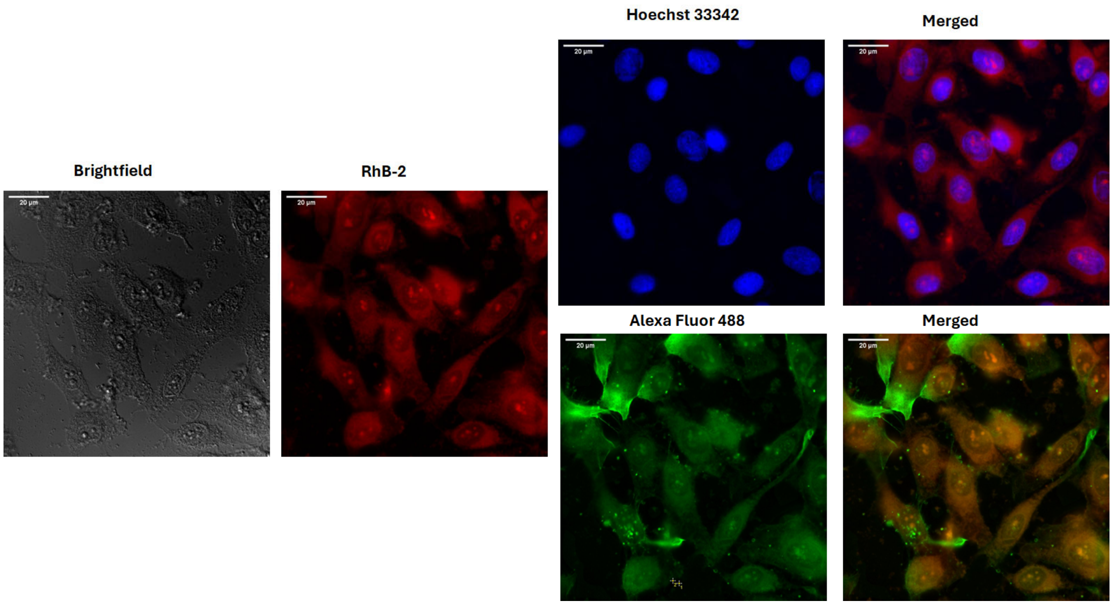

Figure S15. Fluorescence confocal microscopy images for HeLa cells treated after two hours with RhB-2 at their respective IC_{50}. Stains for the nucleus (Hoechst 3322) and cytoplasmic actin (Alexa Fluor 488 alpha SMA) were employed for determining colocalization.

Table S3. Colocalization Analysis. Quantitate colocalization analysis of labeled peptides with cytoplasm and nuclear fluorophores employing JaCoP Image J analysis software.

	RhB-1 / Alexa Fluor 488 SMA	RhB-1 / Hoechst 3322	RhB-2 / Alexa Fluor 488 SMA	RhB-2 / Hoechst 3322
Correlation coefficient	0.444	0.697	0.664	0.488
Manders Coefficient M1 (Red)	0.488	0.962	0.947	0.345
Manders Coefficient M2 (Blue/Green)	0.749	0.746	0.77	0.435

Correlation coefficient: linear correlation of signal Red with either blue or green in the cytofluorogram
M1: Fraction of signal Red colocalizing with either signal Blue or Green
M2: Fraction of either signal Blue or Green colocalizing with signal Red

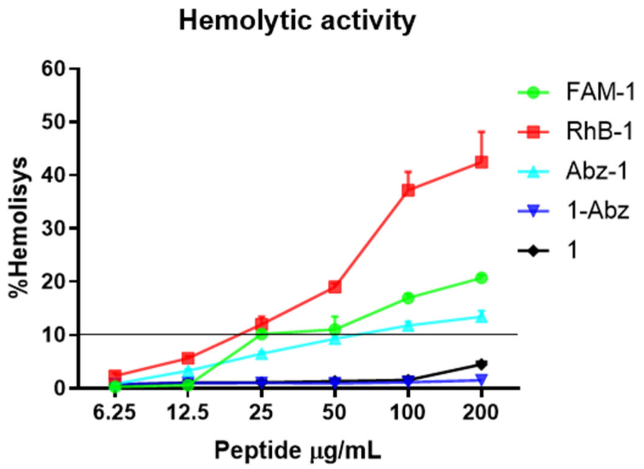

Figure S16. Hemolytic activity of peptides at concentrations of 6.25 to 200 µg/mL, hemolytic activity is dependent on the concentration of the peptide. The black line shows the limit of 10% of hemolysis.

Table S4. The selectivity index corresponds to the ratio between the peptide concentration at 10% hemolysis/IC_{50} in HeLa cells. The therapeutic index corresponds to the ratio between peptide concentration at 10% hemolysis/MIC of the strain with the highest activity.

Code	Sequence	Percentage of hemolysis*	Selectivity index	Therapeutic index
**1**	RWQWRWQWR	1.1%	>3	>8
**1-Abz**	RWQWRWQWR-(2-Abz)	0.9%	NA	>4
**Abz-1**	(2-Abz)-RWQWRWQWR	6.5%	0.7	4
**FAM-1**	FAM-RWQWRWQWR	17.0%	NA	0.25
**RhB-1**	RhB-RWQWRWQWR	19%	0.2	0.5

*Percentage of hemolysis at the lowest MIC concentration. NA: not applicable, Peptides where the IC_{50} was not found

Disclaimer/Publisher's Note: The statements, opinions and data contained in all publications are solely those of the individual author(s) and contributor(s) and not of MDPI and/or the editor(s). MDPI and/or the editor(s) disclaim responsibility for any injury to people or property resulting from any ideas, methods, instructions or products referred to in the content.
